# Higher-order spatiotemporal wave packets with Gouy phase dynamics

**DOI:** 10.1515/nanoph-2025-0508

**Published:** 2025-12-10

**Authors:** Wangke Yu, Yijie Shen

**Affiliations:** 54761 Centre for Disruptive Photonic Technologies, School of Physical and Mathematical Sciences, Nanyang Technological University, Singapore 637371, Singapore; School of Electrical and Electronic Engineering, 54761 Nanyang Technological University, Singapore 639798, Singapore

**Keywords:** spatiotemporal wave packets, structured light, Gouy phase, spatiotemporal revivals (self-healing), ultrashort pulse, phase and group velocity

## Abstract

Spatiotemporal (ST) wave packets constitute a broad class of optical pulses whose spatial and temporal degrees of freedom cannot be treated independently. Such space-time non-separability can induce exotic physical effects such as non-diffraction, non-transverse waves, and sub or superluminal propagation. Here, a higher-order generalised family of ST modes is presented, where modal orders are proposed to enrich their ST structural complexity, analogous to spatial higher-order Gaussian modes. This framework also incorporates spatial eigenmodes and typical ST pulses (e.g., toroidal light pulses) as elementary members. The modal orders are strongly coupled to the Gouy phase, which can unveil anomalous ST Gouy-phase dynamics, including ultrafast cycle-switching evolution, ST self-healing, and sub/super-luminal propagation. We further introduce a stretch parameter that stretches the temporal envelope while keeping the Gouy-phase coefficient unchanged. This stretch invariance decouples pulse duration from modal order, allowing us to tune the few-cycle width without shifting temporal-revival positions or altering the phase/group-velocity laws. Moreover, an approach to analysing the phase velocity and group velocity of the higher-order ST modes is proposed to quantitatively characterise the sub/super-luminal effects. The method is universal for a larger group of complex structured pulses, laying the basis for both fundamental physics and advanced applications in ultrafast optics and structured light.

## 1 Introduction

It has long been a human aspiration to manipulate extreme structures in electromagnetic fields, and spatiotemporally sculpted light pulses have recently attracted significant interest [1], [2], [3]. In particular, the pursuit of ultrashort few-cycle, and even single-cycle, pulses represents a central goal in achieving extremely fast and effcient energy extraction [4], [5]. Also, the generation and steering of exotic structures in few-cycle pulses hold the promise of extending fundamental scientific effects in light-matter interaction [6], [7], [8], nonlinear physics [9], [10], [11], and spin-orbital coupling [12], [13], [14], as well as in a myriad of novel applications in ultrafast microscopy [15], [16], [17], large-capacity communications [18], [19], [20], [21], particle trapping [22], [23], [24], and material machining [25], [26], [27], [28], to name a few. On the other hand, structured pulses inherently exhibit exceptional propagation characteristics compared to conventional waves, challenging the fundamental physical laws and reshaping our understanding of light. For instance, it was reported that structured pulses can show counterintuitive subluminal group velocities in vacuum [29], [30], [31], less than the widely accepted speed of light in vacuum, *c*, a fundamental constant of nature. Soon after, the superluminal propagation of twisted pulses in some special cases was also demonstrated [32], [33]. With the advanced control of spatiotemporal light, more complex and anomalous propagation with arbitrary sub- and super-luminal behaviour of the light pulse has been realized [34], [35], [36], [37], broadening the frontier of modern physics. See also recent reviews and demonstrations on spatiotemporal (ST) wave packets, metrology, and guided-wave implementations [38], [39], [40], [41], [42], [43]. Therefore, people will never stop exploring more generalized extreme electromagnetic pulses, not only enabling unlimited applications, but also as a fundamental scientific endeavour in itself.

For characterization of optical pulses, there are well-established theories provide a family of space-time non-separable solutions of Maxwell’s equations. However, recent studies highlighted the much broader class of ST wave packets, where the spatial and temporal dependence cannot be treated separately. As one of the earliest models of ST non-separable pulses, in 1983, Brittingham proposed the localized solutions of Maxwell’s equations to obtain the focused spatiotemporal wave modes [44]. Soon after, Ziolkowski developed such space-time non-separable solutions to the scalar wave equation with moving complex sources [45], and proposed that a superposition of such pulses leads to finite energy pulses termed “electromagnetic directed-energy pulse trains” (EDEPT) [46]. Special cases of Ziolkowski’s solutions were studied by Hellwarth and Nouchi, who found closed-form expressions that describe focused single-cycle finite-energy solutions to Maxwell’s equations [47]. This family of pulses includes linearly polarised pulses, termed “flying pancakes” (FPs) [48], as well as pulses of toroidal symmetry, termed “flying doughnuts” (FDs) [47]. In the last few decades, the classical FP and FD pulses have become the widely-endorsed representatives of few-cycle solutions for guiding developments of generations and applications of ultrashort pulses. For instance, studies on the linearly polarised FP pulse, including its propagation [48], [49], diffraction [50], [51], and cavity oscillation [50], [52] paved the way for developing the advanced femtosecond few-cycle mode-locked laser [53], [54], [55], [56]. Meanwhile, the toroidally structured FD pulses with more exotic spatial electrodynamics, than the FP pulses, have held more promises of exciting applications particularly in the contexts of non-radiating anapole materials [57], [58], topological information transfer [59], probing ultrafast light–matter interactions [60], and toroidal excitations in matter [61], [62]. It was also recently demonstrated that the FD pulses can be generated by tailored metamaterials which convert traditional few-cycle pulse into an FD pulse [63], [64].

The classical localised ST wave packets discussed above provide strong confinement and directionality, but they usually tie the field structure to the pulse duration. In the spatial domain, we have well-known eigenmode families (such as Hermite–Gaussian (HG) and Laguerre–Gaussian (LG)). A comparable, unified, and programmable family for localised ST pulses has been missing. Here we introduce an analytic higher-order family of FP and FD pules with two decoupled controls. The modal order *α* is the structural dial: it sets the spatiotemporal complexity and fixes the effective Gouy phase coefficient, which in turn determines temporal revivals and cycle switching. The stretch parameter *p* is the duration dial: it lengthens the co-moving envelope without shifting those Gouy-controlled landmarks, smoothly bridging few-cycle pulses toward quasi-CW, beam-like eigenstructures. All fields are given in closed form a single scalar generator lifted by a Hertz potential, and we provide a consistent framework for phase- and group- velocity diagnostics.

### 1.1 Fundamental theories

The focused few-cycle electromagnetic pulses can be described as localized, finite-energy, space-time non-separable solutions to Maxwell’s equations. Among the established approaches for obtaining these exact solutions, the EDEPT method is one of the most widely adopted [46]. The first step is to find a scalar generating function *f*(**r**, *t*) that satisfies the Helmholtz equation:

(1) $$\left(\nabla^{2}-\frac{1}{c^{2}}\frac{\partial^{2}}{\partial t^{2}}\right)f\left(r,t\right)=0,$$

where **r** is the spatial coordinate (which may be in Cartesian coordinates (*x*, *y*, *z*) or cylindrical coordinates (*r*, *θ*, *z*)), *t* is time, $c=1/\sqrt{\varepsilon_{0}\mu_{0}}$ is the speed of light, and *ɛ*_0_ and *μ*_0_ denote the permittivity and permeability of free space. The exact solution for *f*(**r**, *t*) can be obtained using the modified power-spectrum method proposed by Ziolkowski [45], [46]:

(2) $$f\left(r,t\right)=f_{0}\frac{e^{-s/q_{3}}}{\left(q_{1}+i\tau \right){\left(s+q_{2}\right)}^{\alpha }},$$

where *f*_0_ is a normalisation constant, *s* = *r* ^2^/(*q*_1_ + *iτ*) − *iσ*, *τ* = *z* − *ct*, *σ* = *z* + *ct*, and *q*_1_, *q*_2_, *q*_3_ are positive real adjustable parameters with units of length. The dimensionless parameter *α* arises from the finite-energy condition and must satisfy *α* ≥ 1 for the electromagnetic pulse to have finite energy. To obtain exact solutions of Maxwell’s equations for the electric and magnetic fields **E**(**r**, *t*) and **H**(**r**, *t*), we construct a Hertz potential $\Pi \left(r,t\right)=\hat{n}f\left(r,t\right)$ , where $\hat{n}$ is an arbitrary unit vector. The corresponding transverse electric (TE) electromagnetic field can then be derived from this Hertz potential as follows [46]:

(3) $$E\left(r,t\right)=-\mu_{0}\frac{\partial }{\partial t}\nabla \times \Pi ,$$

(4) $$H\left(r,t\right)=\nabla \times \left(\nabla \times \Pi \right).$$

In the classical few-cycle solutions (FP and FD modes), the finite-energy parameter is fixed at *α *= 1, and *q*_3_ is set to an infinite value. Here we derive the closed-form expressions for arbitrary finite-energy parameter *α* ≥ 1, which we refer to as higher-order ST pulses. When the Hertz potential vector is chosen as $\hat{n}=\hat{x}$ , the *α*-dependent closed-form expression of the linearly polarized FP pulses (polarized along the *y* axis) is obtained as follows (see the detailed derivation in the Supplementary Information):

(5) $$\begin{array}{ll} E_{\alpha }^{\left(\text{FP}\right)}\left(r,t\right) & =\alpha \left(\alpha +1\right)f_{0}\sqrt{\frac{\mu_{0}}{\varepsilon_{0}}}{\left(q_{1}+i\tau \right)}^{\alpha -1}\\ & \;\times \frac{{\left(q_{1}+i\tau \right)}^{2}-{\left(q_{2}-i\sigma \right)}^{2}}{{\left[r^{2}+\left(q_{1}+i\tau \right)\left(q_{2}-i\sigma \right)\right]}^{\alpha +2}}. \end{array}$$

For $\hat{n}=\hat{z}$ , we obtain the *α*-dependent azimuthally polarized electric field (see the detailed derivation in the Supplementary Information):

(6) $$\begin{array}{ll} E_{\alpha }^{\left(\text{FD}\right)}\left(r,t\right) & =-\alpha \left(\alpha +1\right)if_{0}\sqrt{\frac{\mu_{0}}{\varepsilon_{0}}}{\left(q_{1}+i\tau \right)}^{\alpha -1}\\ & \;\times \frac{r\left(q_{1}+q_{2}-2ict\right)}{{\left[r^{2}+\left(q_{1}+i\tau \right)\left(q_{2}-i\sigma \right)\right]}^{\alpha +2}}, \end{array}$$

where *q*_1_ and *z*_0_ = *q*_2_/2 determine the wavelength and Rayleigh range.

Under the paraxial limit (*q*_1_ ≪ *q*_2_), we can further reduce it to a local-time amplitude-phase expression with clearer manifestation of beam propagation (see the detailed derivation in the Supplementary Information):

(7) $$E_{\alpha }^{\left(\text{FP}\right)}\left(r,t\right)=\frac{\alpha \left(\alpha +1\right)w_{0}^{\alpha }A_{\alpha }\left(r,t\right)}{2^{\alpha }z_{0}^{\alpha }w^{\alpha }{\left(1+\frac{r^{2}}{2w^{2}}\right)}^{\alpha +2}}{\text{e}}^{\text{i}\left[k_{\alpha }\left(r,t\right)+\alpha \phi \left(z\right)\right]},$$

(8) $$\begin{array}{ll} E_{\alpha }^{\left(\text{FD}\right)}\left(r,t\right) & =\frac{i\alpha \left(\alpha +1\right)w_{0}^{\alpha +1}rA_{\alpha }\left(r,t\right)}{2^{\alpha }z_{0}^{\alpha +1}w^{\alpha +1}{\left(1+\frac{r^{2}}{2w^{2}}\right)}^{\alpha +2}},\\ & \;\times {\text{e}}^{\text{i}\left[k_{\alpha }\left(r,t\right)+\left(\alpha +1\right)\phi \left(z\right)\right]} \end{array}$$

where, $w\left(z\right)=w_{0}\sqrt{{\left[1+\left(\frac{z}{z_{0}}\right)\right]}^{2}}$ and $w_{0}^{2}=q_{1}z_{0}$ decide the beam radius profile and beam waist size, $\phi \left(z\right)=\tan^{-1}\left(\frac{z}{z_{0}}\right)$ is the Gouy phase. The generalized local-time amplitude and wavenumber are defined by $A_{\alpha }\left(r,t\right)=-\frac{f_{0}\mu_{0}c{\left(q_{1}^{2}+\tau^{2}\right)}^{\left(\alpha -1\right)/2}}{q_{1}^{\alpha +2}{\left(T^{2}+1\right)}^{\left(\alpha +2\right)/2}}$ and $k_{\alpha }\left(r,t\right)=\left(\alpha -1\right)\tan^{-1}\left(\frac{\tau }{q_{1}}\right)+\left(\alpha +2\right)\tan^{-1}T$ . Specially when *α* = 1, the higher-order FP and FD modes reduce to the fundamental FP and FD pulses [47], [48]. We note that it is reasonable to name the solutions of $E_{\alpha }^{\left(\text{FP}\right)}\left(r,t\right)$ and $E_{\alpha }^{\left(\text{FD}\right)}\left(r,t\right)$ as higher-order FP and FD pulses, because they have a structure extremely similar to the classical spatial higher-order modes, such as the higher-order HG and LG modes:

(9) $$E_{m,n}^{\left(\text{HG}\right)}\left(r\right)=\frac{c_{m,n}}{w}H_{m}\left(\tilde{x}\right)H_{n}\left(\tilde{y}\right){\text{e}}^{-\frac{{\tilde{r}}^{2}}{2}}{\text{e}}^{\text{i}\left[k_{\lambda }\tilde{z}+\left(m+n+1\right)\phi \left(z\right)\right]},$$

(10) $$E_{p,\ell }^{\left(\text{LG}\right)}\left(r\right)=\frac{c_{p,\ell }}{w}{\tilde{r}}^{\left|\ell \right|}L_{p}^{\left|\ell \right|}\left(\tilde{r}\right){\text{e}}^{-\frac{{\tilde{r}}^{2}}{2}}{\text{e}}^{-\text{i}\ell \theta }{\text{e}}^{\text{i}\left[k_{\lambda }\tilde{z}+\left(2p+|\ell |+1\right)\phi \left(z\right)\right]},$$

where $\tilde{u}=\frac{\sqrt{2}u}{w\left(z\right)}$ (*u* = *x*, *y*, *r*), $\tilde{z}=z+\frac{r^{2}}{2R\left(z\right)}$ , and $k_{\lambda }=\frac{2\pi }{\lambda }$ is the wavenumber; *H*_*m*_ and $L_{p}^{\ell }$ denote the Hermite and generalized Laguerre polynomials, respectively, and *c*_*m*,*n*_ or *c*_*p*,_ *_ℓ_* is the normalized coefficient. Clearly, the order *α* in the higher-order FP and FD pulses [Eqs. (7) and (8)] follows the same principle as the spatial mode orders (*m*, *n*) and (*p*, *ℓ*) in the HG and LG modes [Eqs. (9) and (10)], in the sense that higher order leads to more intricate field patterns and larger Gouy phase shifts. However, an important difference is that the mode order in HG and LG beams are restricted to non-negative integers, whereas the order *α *in higher-order FP and FD pulses can take any real value larger than one, i.e. *α* ≥ 1, owing to the finite-energy condition.

**Figure 1:**
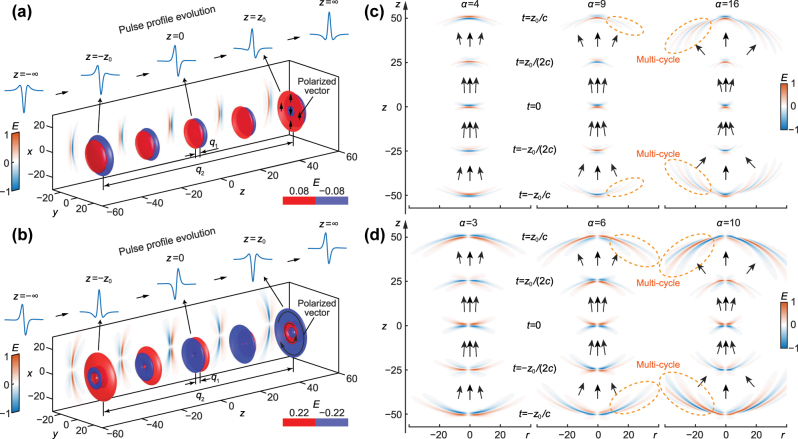
Spatiotemporal evolutions of the fundamental FP and FD pulses: (a), (b) iso-amplitude surfaces for the electric fields *E* ^(FP)^(**r**, *t*) and *E* ^(FD)^(**r**, *t*), plotted at amplitude levels *E* = ±0.08 and *E* = ±0.22, respectively, with *q*_2_ = 100*q*. The fields are shown at *t* = 0, ±*z*_0_/(2*c*), and ±*z*_0_/*c*, together with temporal profiles at spatial positions, *z* = 0, ±*z*_0_, and ±∞, and the corresponding *x*–*z* maps of instantaneous electric fields. Spatiotemporal evolutions of higher-order FP and FD pulses: (c), (d) the *r*–*z* distributions for the electric fields $E_{\alpha }^{\left(\text{FP}\right)}\left(r,t\right)$ with *α* = 4, 9, and 16, and $E_{\alpha }^{\left(\text{FD}\right)}\left(r,t\right)$ with *α* = 3, 6, and 10, at *t* = 0, ±*z*_0_/(2*c*), and ±*z*_0_/*c. *Multi-cycle structures are highlighted with dashed lines. The spatial coordinate is normalised by *q*_1_. For detailed dynamics of pulses with various orders *α*, see Video 1 and Video 2 in Supplementary Materials.

When *α* = 1, the real and imaginary parts of both *E* ^(FP)^(**r**, *t*) and *E* ^(FD)^(**r**, *t*) correspond to two distict pulses, i.e. the focused single-cycle pulse and the focused $1\frac{1}{2}$ -cycle pulse (depending on whether the pulse is single-cycle or $1\frac{1}{2}$ -cycle at focus), and the single-cycle and $1\frac{1}{2}$ -cycle structures are reshaped during propagation for both cases. In contrast to the FP mode, the FD has a more complex amplitude pattern and possesses a Gouy phase that is twice that of the FP. In this regard, the FD can be viewed as a higher-order counterpart of the FP, much like how higher-order spatial modes feature more complicated patterns and larger Gouy phase shifts compared to the fundamental mode [65]. The Gouy phase is the most crucial factor governing the temporal evolution of few-cycle pulses [49]. Consequently, the FP and FD modes exhibit distinct spatiotemporal behaviours. The corresponding simulations for the focused single-cycle FP and FD pulses are shown in Figure 1(a) and (b), respectively. For the FP pulse, a $1\frac{1}{2}$ -cycle structure from negative infinity position gradually evolves into the single-cycle at focus, then evolves into another conjugate $1\frac{1}{2}$ -cycle structure at positive infinity position. For the FD pulse, the evolution has a stronger space-time non-separability because its Gouy phase is twice than that of the FP pulse, where the negative infinity position shows a single-cycle profile, it already evolves into the $1\frac{1}{2}$ -cycle at negative Rayleigh range, next evolves into the conjugate single-cycle at focus, and then to the conjugate $1\frac{1}{2}$ -cycle at positive Rayleigh range, finally to the single-cycle at positive infinity position same as the one located at negative infinity. Thus the doubled Gouy phase in the FD reveals the faster temporal reshaping effects.

### 1.2 Envelope stretching via parameter *p*: exact forms and diagnostics

Within the EDEPT framework, a spectral phase mask

$$S_{p}\left(a\right)=\underset{m}{\sum }w_{m}\;e^{-ia\Delta_{m}},\;\underset{m}{\sum }w_{m}=1,$$

acts as a coherent superposition of baseline solutions, each term acquiring a shift *σ* → *σ* − Δ_*m*_. Our *p-*stretch EDEPT formulation uses the kernel ${\text{e}}^{\text{i}\alpha \Delta_{m}}$ . For forward propagation along the +*z-*direction, we make the substitution *σ* = −*τ* (equivalently, *S* = *q*_2_ − *iσ* to *S* = *q*_2_ + *iτ*). This replacement leaves the results of the FD/FP Gouy-phase coefficient unchanged; see the Supplementary H, “Forward propagation”. We define

$$\begin{array}{c} A:=q_{1}+i\tau ,\;\;S_{m}:=q_{2}-i\left(\sigma -\Delta_{m}\right),\\ B_{m}:=\rho^{2}+A\;S_{m},\;\;\rho \equiv r. \end{array}$$

The exact FP/FD electric fields with stretch parameter *p* are then given by:

(11) $$\begin{array}{ll} E_{\alpha ,p}^{\left(FP\right)}\left(r,t\right) & =\sum_{m}w_{m}\;\left[-\alpha \left(\alpha +1\right)f_{0}\sqrt{\mu_{0}/\varepsilon_{0}}\;A^{\alpha -1}\right.\;\left.\frac{A^{2}-S_{m}^{2}}{B_{m}^{\alpha +2}}\right], \end{array}$$

(12) $$\begin{array}{ll} E_{\alpha ,p}^{\left(FD\right)}\left(r,t\right) & =\sum_{m}w_{m}\;\left[+\alpha \left(\alpha +1\right)if_{0}\sqrt{\mu_{0}/\varepsilon_{0}}\;A^{\alpha -1}\right.\;\left.\frac{\rho \;\left(A+S_{m}\right)}{B_{m}^{\alpha +2}}\right]. \end{array}$$

**Figure 2:**
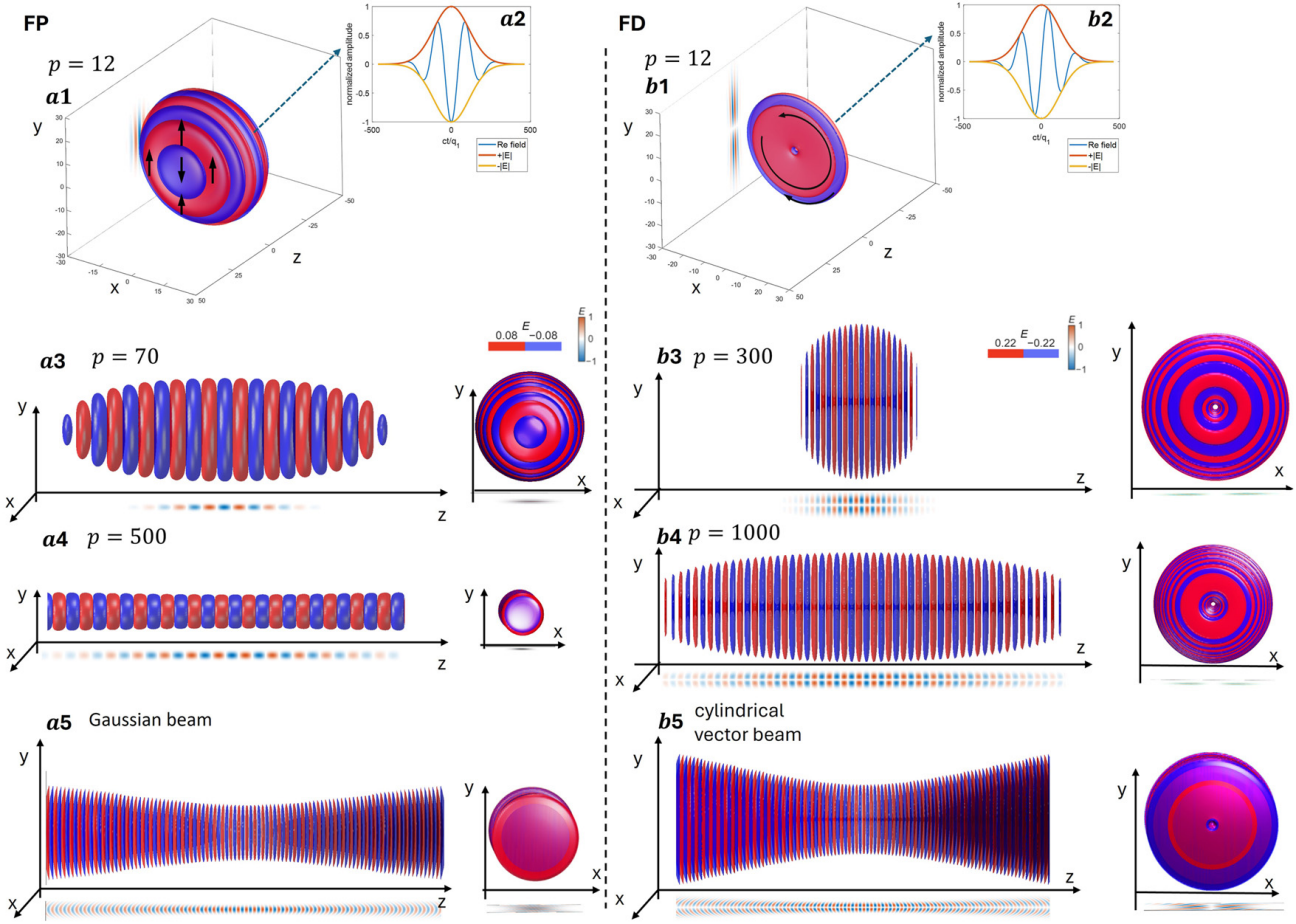
Iso-amplitude evolution of FP/FD pulses under p-stretching and in the beam-limit regime. Left column: FP; right column: FD. (a1, b1) Three-dimensional iso-amplitude surfaces of the electric field for a representative stretch *p* = 12; arrows indicate the local polarization (FP: linear *y*; FD: azimuthal). (a2, b2) Insets: time traces for the same parameters [Re *E* together with ±|*E*|]. (a3, a4) Three-dimensional iso-amplitude surfaces of the FP pulses for *p* = 70 and *p* = 500. As *p* increases, the iso-amplitude slices form an increasingly dense stack of half-cycle sheets along the local-time direction, corresponding to an extended temporal envelope. (a5) In the large-*p* limit, the FP pulse approach a linearly polarized Gaussian beam; (b3, b4) Three-dimensional iso-amplitude surfaces of the FD pulse for *p* = 300 and *p* = 1,000. As *p* increases, the iso-amplitude slices similarly become more densely stacked, leading to a longer temporal envelope. (b5) In the large-*p* limit, the FD pulse approaches a cylindrical-vector (azimuthally polarized) beam. Red/blue colors encode the sign of the instantaneous electric field; the color bars indicate the normalized iso-amplitude levels used in the renderings. Supplementary Videos 1 and 2 further show the full spatiotemporal evolution of higher-order FP and FD pulses for the different values of the stretch parameter *p*.

The parameter *p* controls the length of the temporal envelope through {Δ_*m*_, *w*_*m*_} while leaving the Gouy coefficient unchanged (FD exceeds the FP by one unit for the same *α*). Figure 2 visualizes how the *p*-stretch transforms the pulse morphology and drives it towards the beam limit, illustrated using iso-amplitude renderings. For a moderate stretch (*p* = 12), panels (a1, b1) show the three-dimensional iso-amplitude surfaces of the instantaneous electric field, together with the expected polarization distributions (linear *y* for FP and azimuthal for FD); the insets (a2, b2) display the corresponding time traces [Re *E* with ±|*E*|]. As *p* increases (FP: a3 → a4; FD: b3 → b4), the iso-amplitude slices form an increasingly dense stack of half-cycle sheets along the local-time direction. The temporal envelope becomes longer with increasing *p*, whereas the transverse profile set by *q*_1_ and *q*_2_ remains unchanged; the axial range displayed is kept fixed for comparison. Panels (a5, b5) highlight the large-*p* limit: the FP continuously approaches a linearly polarised Gaussian beam, while the FD approaches a cylindrical-vector (azimuthally polarised) beam. These observations are fully consistent with the *p*-stretch acting solely on the temporal degree of freedom (envelope lengthening), while leaving the modal polarisation and spatial symmetries unchanged. The de-chirped envelope shows one-sided spectra (Supplementary Information, Figures S2 and S3) that narrow approximately as $\Delta \Omega \propto \frac{1}{\sqrt{p}}$ , quantitatively confirming the trasition toward a quasi-CW bridge while the Gouy coefficient remains unchanged. For detailed derivations, see the Supplementary Information.

### 1.3 Spatiotemporal evolution of ST pulses of higher order

The spatiotemporal structures for the pulses of higher order FP (*α* = 4, 9, 16) and FD (*α* = 3, 6, 10) are demonstrated in Figure 1(c) and (d), respectively, where some basic properties can be observed. For the spatial property, a higher-order pulsed mode always has a more serious divergence, this principle is same as that of conventional higher-order spatial modes. Regarding temporal profile evolution, higher order-pulses typically exhibit faster temporal reshaping; for example, the 4th-order FP and 3rd-order FD pulses, which possess a fourfold Gouy-phase shift, undergo double cycle switching events within a single Rayleigh range (it from the single-cycle evolves to the $1\frac{1}{2}$ -cycle and then to the conjugate single-cycle at focus). As the order increases further, the switch between single- and $1\frac{1}{2}$ -cycle structures becomes more frequent, with stronger space-time non-separability. Moreover, for very high oeders, unconventional multi-cycle structures emerge, as marked in Figure 1(c) and (d). A striking pulse evolution occurs in this regime, where a multi-cycle structure can be evolved into a single-cycle at the focus. In addition, as the order *α *increases, the HOSTP shows stronger localization, with the effective energy becoming more tightly confined in the transverse direction at focus.

**Figure 3:**
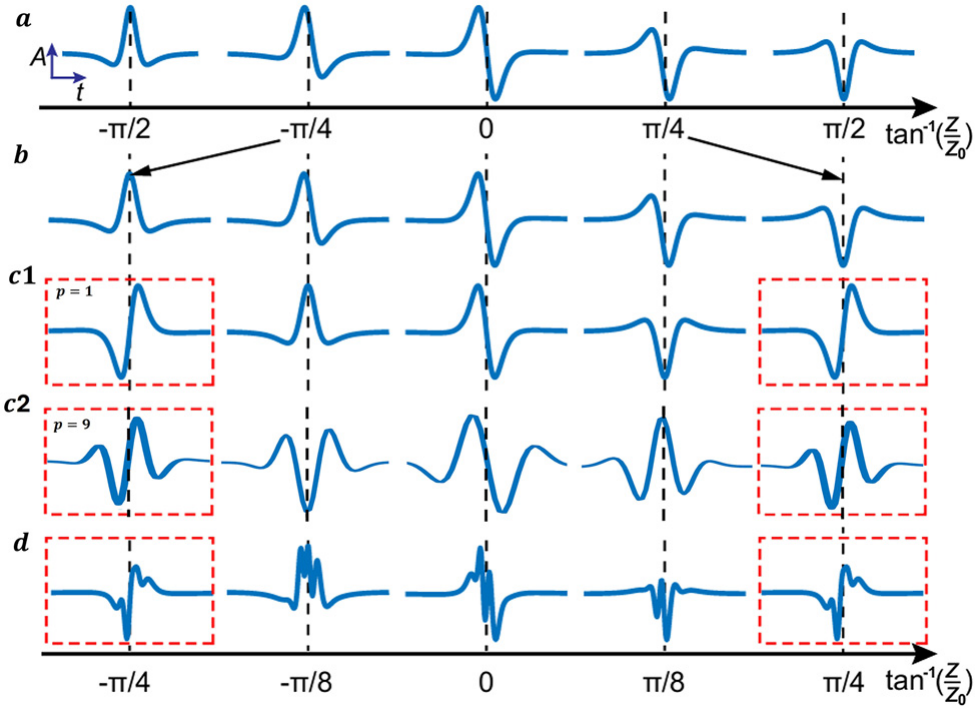
Spatiotemporal revivals and *p*-stretch invariance. (a) Fundamental FP; (b) fundamental FD; (c) 3rd-order FD (*α* = 3); (d) a superposed state $E_{\alpha =3}^{\left(FD\right)}+E_{\alpha =7}^{\left(FD\right)}+E_{\alpha =11}^{\left(FD\right)}$ . Vertical dashed lines indicate sampling positions *ϕ* = tan^−1^(*z*/*z*_0_). In (c), two rows with *p* = 1 (top) and *p* = 9 (bottom) are shown. The red dashed boxes mark the revival positions at *ϕ* = ±*π*/4; the boxed waveforms for *p* = 1 and *p* = 9 coincide, demonstrating that although *p* stretches the temporal envelope, the Gouy-phase coefficient and the revival locations remain unchanged (*p*-stretch invariance). All superpositions use equal energy components: each field is locally time-normalized (FP at *r* = 0; FD at the ring maximum) before summation.

Pattern revivals driven by fractional Gouy phases are by now a well-established feature of structured beams. When a transverse field is written as a superposition of spatial eigenmodes with different Gouy coefficients, the accumulated phase differences ΔΨ(*z*) = (*N*_1_ − *N*_2_) *ϕ*(*z*) may periodically re-synchronise, reconstructing the initial intensity pattern at discrete propagation planes [66], [67]. In our HOSTP family this mechanism is carried over from the purely spatial domain to the spatiotemporal domain. The order index *α* plays the role analogous to the transverse mode order: it fixes the Gouy-phase coefficient *C*_*α*_ [with *C*_*α*_ = *α* for FP and *C*_*α*_ = *α* + 1 for FD], so that the longitudinal phase advance is *C*_*α*_ *ϕ*(*z*). During propagation the local spatiotemporal profile undergoes strong reshaping, yet at propagation distances satisfying

(13) $$C_{\alpha }\;\phi \left(z_{q}\right)=2\pi q,\;q\in Z,$$

the relative Gouy phases return to their initial values and the few-cycle temporal waveform revives. Figure 3(c1)–(d) illustrate these spatiotemporal revivals for both the HOSTP and its multi-order superpositions. In this sense, our construction generalises the well-known fractional Gouy-phase pattern revival from transverse beam profiles to fully spatiotemporal wave packets: the same phase mechanism that reconstructs a spatial pattern can now rebuild the spatiotemporal shape of an ultrafast pulse. Figure 3(a) and (b) show the temporal-profile evolutions of the fundamental FP pulse and FD pulses, also displayed in Figure 1(a) and (b). In these fundamental cases, the pulse changes throughout the propagation region, and no temporal-profile revival occurs. However, we can observe that the FD evolves more rapidly: its waveform changes from a $1\frac{1}{2}$ -cycle structure to its opposite $1\frac{1}{2}$ -cycle structure over a Rayleigh range, but the FP pulse requires propagation across the entire region to undergo the same transition. Figure 3(c) shows the case of a higher-order FP pulse with *α* = 3 (equivalently, a higher-order FP pulse of order *α* = 4), where the waveform returns to its initial shape within one Rayleigh range. In general, the higher the order, the faster the revival, in accordance with Eq. (13). The number of admissible *q-*values determins how many revivals appear within the propagation domain. You can notice that in Figure 3(c), two additional rows with *p* = 1 and *p* = 8 explicitly test the effect of envelope stretching. The boxed profiles at *ϕ* = ±*π*/4 for *p* = 1 and *p* = 8 confirm that increasing *p* only lengthens the temporal envelope (larger FWHM) whereas the Gouy-phase coefficient *C*_*α*_ (FD larger than FP by one for the same *α*) and the corresponding revival locations *C*_*α*_ tan^−1^(*z*/*z*_0_) remain unchanged. We also analyse complex superposed pulses with fractional Gouy phase effect. In this case, the revival repeats only if all Gouy phases of all pulse components resynchronize along propagation. For instance, for the superposed pulse $E_{\alpha =3}^{\left(\text{FD}\right)}+E_{\alpha =7}^{\left(\text{FD}\right)}+E_{\alpha =11}^{\left(\text{FD}\right)}$ , all Gouy phases are re-synchronized only at the largest revival distance determined by $E_{\alpha =3}^{\left(\text{FD}\right)}$ . At this distance, the full complex pulse profile revives after one Rayleigh range, as shown in Figure 3(d).

### 1.4 Phase velocity

The HOSTP has a more complex spatiotemporal structure than a conventional pulse, such that both its amplitude envelope and phase are space-time non-separable, i.e. *E*(**r**, *t*) = *A*(**r**, *t*)e^i*φ*(**r**,*t*)^. We evaluate the phase velocity by adopting the original definition that is corresponds to the speed of a moving isophase surface. The phase is space-time-dependent, so that the conventional calculation of phase velocity would be invalid. Based on Eqs. (7) and (8), we can find the space-time-dependent phase expressions for the HOSTP as:

(14) $$\varphi_{\alpha }^{\left(\text{FP}\right)}\left(r,t\right)=i\left[k_{\alpha }\left(r,t\right)+\alpha \phi \left(z\right)\right],$$

(15) $$\varphi_{\alpha }^{\left(\text{FD}\right)}\left(r,t\right)=i\left[k_{\alpha }\left(r,t\right)+\left(\alpha +1\right)\phi \left(z\right)\right].$$

Then, the isophase surface can be determined by the equation of *φ*(**r**, *t*) = *C* where *C* is an arbitrary constant. Next, we take the differential of the isophase surface equation *δφ*(**r**, *t*) = 0 with respect to the variables *z* and *t*, and the local velocity of the motion of the isophase surface can be derived by *v*_p_(*r*, *z*) = *δz*/*δt*, the results of which for the HOSTP are given as (see the detailed derivation in the Supplementary Information E):

(16) $$v_{\text{p}}^{\left(\text{FP}\right)}\left(r,z\right)=\frac{c}{1-\frac{\alpha }{z_{0}}\frac{1}{1+{\left(z/z_{0}\right)}^{2}}/\left[\frac{3}{q_{1}}-\frac{\left(\alpha +2\right)r^{2}}{2q_{1}w^{2}}\right]},$$

(17) $$v_{\text{p}}^{\left(\text{FD}\right)}\left(r,z\right)=\frac{c}{1-\frac{\alpha +1}{z_{0}}\frac{1}{1+{\left(z/z_{0}\right)}^{2}}/\left[\frac{3}{q_{1}}-\frac{\left(\alpha +2\right)r^{2}}{2q_{1}w^{2}}\right]}.$$

**Figure 4:**
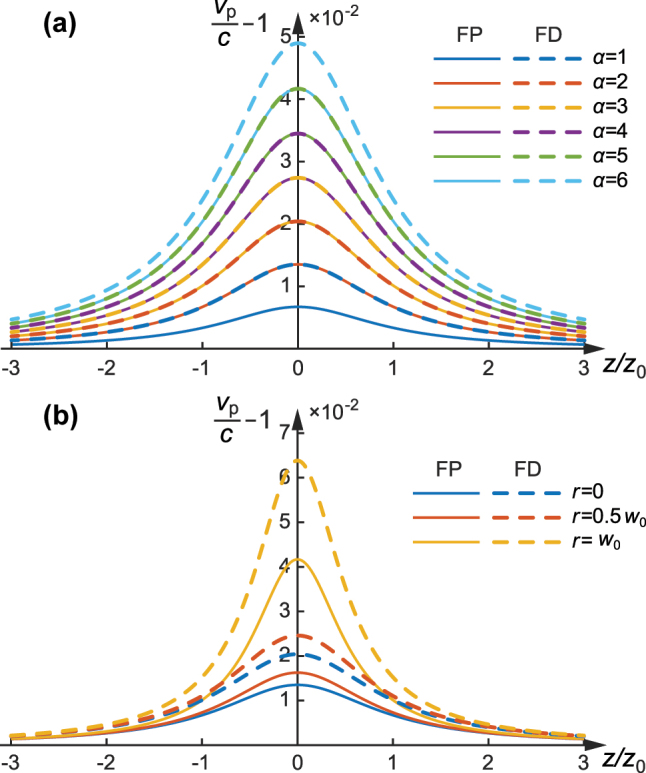
Phase velocity of the HOSTP: (a) on-axis phase velocity distributions versus propagation distance for higher-order FP (solid) and FD (dashed) pulses with order indices *α* from 1 to 6; (b) phase velocity distributions at off-axis positions from *r* = 0 to *r* = *w*_0_ versus propagation distance for the 2nd-order (*α* = 2) FP (solid) and FD (dashed) pulses.

From Eqs. (16) and (17), the only difference between the two phase velocities lies in the coefficients of the denominator terms, *α* and *α* + 1 for the FD and FD pulses, that directly arises from the different Gouy phase *αϕ*(*z*) and (*α* + 1)*ϕ*(*z*), respectively. Thus the (*α* + 1)-order FP pulse has the same phase velocity distribution as the *α*-order FD pulse. That explains why the 4th-order FP and 3rd-order FD pulses shown in the Figure 1(c) and (d) has similar cycle-switching evolution (i.e. they both evolve from a single-cycle to $1\frac{1}{2}$ -cycle and then to single-cycle in the propagation from position of Rayleigh range to the focus). Another conclusion is that the phase velocity is always superluminal (*v*_p_ > *c*), which is consistent with the cycle-switching phenomena that keep a forward direction as observed above. The on-axis (*r* = 0) phase velocity distributions upon propagation of various-order FP nd FD pulses are plotted in Figure 4(a), as *v*_p_/*c* − 1 to highlight the deviation from the speed of light in vacuum. It demonstrates that the phase velocity always reaches the maximum at focus (*z* = 0) and decreases with the propagation position away from the focus. And the phase velocity is always increasing with the increasing of order index *α*. Due to the complex transverse structure of HOSTP, it is meaningful to study the off-axis phase velocity, the phase velocity distributions at various off-axis displacements (from *r* = 0 to *r* = *w*_0_) of the 2nd-order (*α* = 2) FP and FD pulses are shown in Figure 4(b). It reveals that the phase velocity is increasing at a location with increasing off-axis displacement, which explains why there is more rapid and complex cycle-switching structure at the off-axis wing regions of a HOSTP as observed hereinbefore. Phase velocity maps show iso-phase pattern speeds and can exceed c; they do not represent energy or information flow (see Supplementary Information Section H7).

### 1.5 Group velocity

The group velocity is important because it reveals the effective speed of energy transfer. There are various definitions and formulas for evaluating group velocity of structured pulse based on different perspectives [29], [30], [31], [32], [33], [34], [35], [36], however, many of these approaches are difficult to apply to our HOSTP due to its more complex space-time non-separable structure than prior pulses. Here we revisit the original definition of group velocity as the speed of the moving amplitude envelope of a pulse, while there is still a debate on how to evaluate the travelling of amplitude envelope. Our method begins by identifying the centroid surface of the amplitude envelope and then solving the travelling speed of the centroid surface. The expression of the space-time-dependent amplitude envelope of HOSTP can be obtained after simplification (see the detailed derivation in the Supplementary Information):

(18) $$A_{\alpha }^{\left(\text{FP}\right)}\left(r,t\right)=\frac{{\left[q_{1}^{2}+{\left(z-ct\right)}^{2}\right]}^{\left(\alpha -1\right)/2}}{w^{\alpha }{\left[{\left(z-ct+\frac{r^{2}}{2R}\right)}^{2}+q_{1}^{2}{\left(1+\frac{r^{2}}{2w^{2}}\right)}^{2}\right]}^{\left(\alpha +2\right)/2}},$$

(19) $$A_{\alpha }^{\left(\text{FD}\right)}\left(r,t\right)=\frac{r{\left[q_{1}^{2}+{\left(z-ct\right)}^{2}\right]}^{\left(\alpha -1\right)/2}}{w^{\alpha +1}{\left[{\left(z-ct+\frac{r^{2}}{2R}\right)}^{2}+q_{1}^{2}{\left(1+\frac{r^{2}}{2w^{2}}\right)}^{2}\right]}^{\left(\alpha +2\right)/2}}.$$

Based on Eqs. (18) and (19), the sole difference between the amplitude expressions of FP and FD pulses is a factor of *r*/*w*, i.e. $A_{\alpha }^{\left(\text{FD}\right)}=\frac{r}{w}A_{\alpha }^{\left(\text{FP}\right)}$ , this factor is much more slowly varying than the main term, thus the centroid of two envelopes of FP and FD pulses is basically identical for the same order index *α*. The next step is to determine the centroid surface of the envelope. For the fundamental (*α* = 1) FP and FD pulses, the centroid trajectory can be readily obtained as:

(20) $$F\left(r,t\right)=z-ct+\frac{r^{2}}{2R}=0$$

because on this trajectory the envelope attains its maximum (the denominator term attains its minimum). However, for the higher-order (*α* > 1) cases, the profile of the envelope is more curved and the centroid surface deviates from Eq. (20). Here we use the perturbation method to obtain the centroid surface *F*_*α*_(**r**, *t*) = 0 which is applicable for the higher-order pulse:

(21) $$F_{\alpha }\left(r,t\right)=z-ct+\frac{r^{2}}{2R}+d_{\alpha }\left(r\right)=0,$$

where the perturbation displacement is given by the Taylor-series expansions:

(22) $${\left.d_{\alpha }\left(r\right)=d_{\alpha }^{\prime }\right|}_{r^{2}=0}r^{2}+\frac{{\left.d_{\alpha }^{\prime \prime }\right|}_{r^{2}=0}}{2}r^{4}+\cdots$$

The first nonzero term in the Taylor expansion is propotional to *r* ^2^, owing to the even symmetry. Higher-order cases (*α > *1) require more Taylor coefficients to maintain sufficient accuracy. Numerical simulations shows that retaining only the first term already provides an accurate description of the pulse envelope location for orders up to *α* = 10 (see the Supplementary Information F).

To obtain the propagation velocity of the amplitude envelope, we take the differential of the centroid surface equation *δF*_*α*_(**r**, *t*) = 0 with respect to *z* and *t,* and solve for the group velocity *v*_g_ = *δz*/*δt.* The resulting expressions are given below (see the detailed derivation in the Supplementary Information F):

(23) $$v_{\text{g}}^{\left(\alpha \right)}\left(r,z\right)=\frac{c}{1-\frac{\alpha +2}{6}\frac{z^{2}-z_{0}^{2}}{z^{2}+z_{0}^{2}}r^{2}}.$$

Here $v_{g}^{\left(\alpha \right)}\left(r,z\right)$ is interpreted as the centroid velocity of the amplitude envelope. It is obtained by tracking the centroid surface *F*_*α*_(**r**, *t*) = 0 derived from Eqs. (18) and (19) [cf. Eqs. (20) and (21)]. As shown in the Supplementary Information, Section H7, we also derive a complementary expression for the group velocity based on the local-time variable *T*(*r*, *z*, *t*) defined through the phase parametrisation. That construction leads to a peak velocity:

(24) $$v_{\text{g}}^{\text{peak}}\left(r,z\right)=\frac{c}{1-\frac{r^{2}}{2}\frac{R^{\prime }\left(z\right)}{R^{2}\left(z\right)}},$$

which is independent of the modal order *α* and of the stretch parameter *p* [see the Supplementary Information, Section H, Eq. (H26)]. For the fundamental case *α* = 1 the envelope is symmetric in time and the centroid surface coincides with the *T* = 0 level set, so Eq. (23) reduces exactly to Eq. (24). For higher orders *α* > 1 the envelope becomes slightly skewed and the centroid is displaced relative to the peak; the *α*-dependence in Eq. (23) therefore quantifies a small correction to the universal law Eq. (24) and reflects changes in the envelope shape rather than in the underlying energy-flow speed. Numerical checks (see the Supplementary Information, Section F).

**Figure 5:**
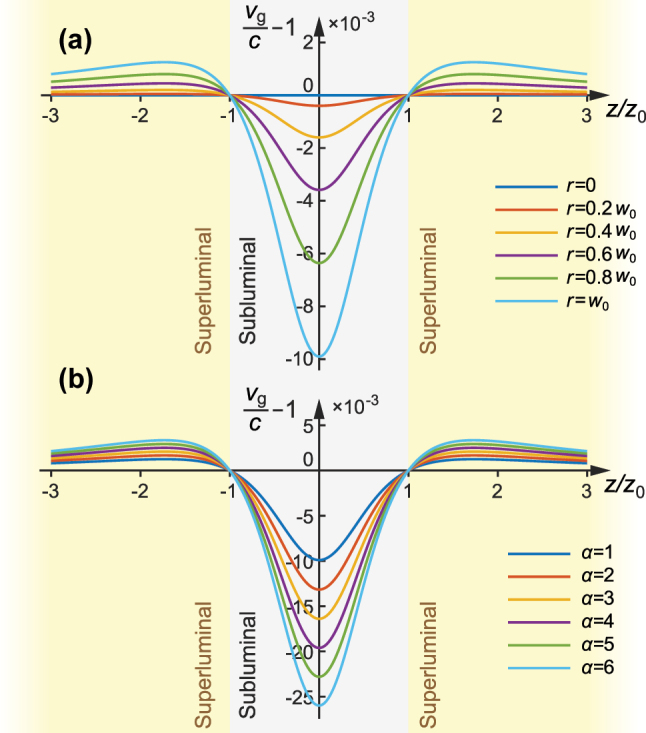
Group velocity of the HOSTP: (a) group velocity distributions at off-axis positions *r* = 0 to *r* = *w*_0_ versus propagation distance for the fundamental FP and FD pulses; (b) group velocity distributions at the off-axis position *r* = *w*_0_ for HOSTP pulses with order indices *α* from 1 to 6. Gray and yellow regions indicate subluminal and superluminal group velocity domains, respectively.

Figure 5(a) shows the group velocity distribution for the fundamental FP and FD pulse on various off-axis displacements from *r* = 0 and *r* = *w*_0_. For the on-axis (*r* = 0) case, the group velocity shows a constant *c*, corresponding to the conventional speed of light in vacuum. While the off-axis displacement is increasing, the group velocity shows a distribution with propagation distance. When the propagation is within the Rayleigh range, the group velocity is subluminal, and the travelling speed reaches the maximum at the focus and decreases to the constant *c* with the propagation distance up to Rayleigh length. When the propagation is beyond the Rayleigh range, the group velocity is superluminal, and the speed is first increasing and then decreasing approaching the limit of *c* with the propagation distance from Rayleigh length to infinity. The larger the off-axis displacement the more serious the deviation from *c* the group velocity distribution is. Such an abnormal distribution is induced by the spatiotemporally curved structure of pulse envelope upon propagation. The wavefront of HOSTP is approximately flat when the propagation is at focus and at the infinity (approaches a spherical wavefront with a very large radius, so it is locally almost planar), and mostly curved at the position of Rayleigh length. We note that such abnormal distribution has been studied in prior structured pulses [32], [35]. Here we demonstrate that a similar behaviour also appears in our HOSTP under the new definition of group velocity. The different group velocity profiles at *r* = *w*_0_ for HOSTP with various orders *α* are shown in Figure 5(b). For higher orders, the off-axis group velocity deviates more strongly from *c*, while the overall pattern of subluminal and superluminal regions remains the same.

Another conclusion is that the value of group velocity is overall smaller than the phase velocity (*v*_p_/*c* − 1 is at 10^−2^ level while *v*_g_/*c* − 1 is at 10^−3^ level), in other words, the phase travelling is always faster than the amplitude envelope travelling, which interprets the phenomena of HOSTP that the cycle-switching keeps forward upon propagation.

## 2 Discussions

We propose and demonstrate a generalized family of focused few-cycle solutions to Maxwell’s equations, namely, higher-order spatiotemporal pulses (HOSTP), which extend the classical few-cycle FP and FD modes. We present local time amplitude-phase expressions for HOSTP; unlike conventional few-cycle pulses, both the amplitude and the phase are spatiotemporally non-separable, enabling a broad range of novel spatiotemporal structures. The family exhibits clear higher-order properties analogous to spatial higher-order modes (more complex amplitude patterns and multiple Gouy-phase accumulations with increasing order), yet with genuinely spatiotemporal extensions. Notably, we observe a strongly non-separable evolution in which a multi-cycle structure evolves into a single-cycle profile at focus, breaking the conventional “fixed cycle-number” description and opening new directions for ultrafast structured light. Within the exact EDEPT construction, a stretch parameter *p*, implemented through a spectral, phase mask that applies the term-wise shift *σ* → *σ* − Δ_*m*_, is used to stretche the temporal envelope while leaving the Gouy-phase coefficient unchanged (FD exceeds FP by one unit for the same *α*). Consequently, spatiotemporal-revival locations governed by *C*_*α*_ tan^−1^(*z*/*z*_0_) remain invariant with respect to *p*, allowing pulse width to be tuned independently of modal order *α*; this follows from the exact *p*-dependent FP/FD forms and the Gouy-phase coefficient analysis in the text and in the Supplementary Information.

To characterise such a complex ST structure, we develop a unified approach to analysing the phase velocity and group velocity of structured pulses. The superluminal phase velocity accounts for the persistent forward cycle-switching, whereas the group velocity exhibits both subluminal and superluminal regions depending on transverse position and propagation distance, an expected feature of structured pulses that does not violate special relativity or causality. The same methodology applies to radial dynamics by taking differentials with respect to *r* and *t* in the iso-phase or centroid-surface equations; in most propagation scenarios, radial-velocity components are much smaller than the longitudinal component and are therefore omitted for brevity.

Because HOSTP introduce additional degrees of freedom for general ST waves, here we have illustrated spatiotemporal evolutions and topologies under representative conditions. Systematic links between broader parameter sets and the resulting dynamics, including tightly focused few-cycle pulses and vortex few-cycle pulses carrying orbital angular momentum, will be the focus of future studies.

An especially promising direction is the exploration of complex spatiotemporal skyrmions within the HOSTP framework . Most optical skyrmions reported so far have been constructed in spatial domains, where the skyrmion topology is encoded in a two-dimensional parameter space such as (*x*, *y*) or (*k*_*x*_, *k*_*y*_) [68], [69]. Very recently, optical spatiotemporal skyrmions have also been demonstrated [70]. In contrast, the HOSTP family provides fully Maxwell-consistent, finite-energy wave packets whose amplitude and phase are intrinsically space-time non-separable. The modal order dial *α *controls multi-cycle structure and Gouy-phase-driven revivals, while the stretch dial *p *sets the temporal extent without disturbing these Gouy landmarks. This combination naturally opens a route to embed skyrmion-like textures in mixed space-time manifolds, for example by mapping the skyrmion order parameter onto (*r*, *z*, *t*) or (*x*, *y*, *t*) through the vector field of higher-order FP/FD modes. In such scenarios the skyrmion core and its surrounding shells can be made to breathe, translate, or revive along propagation in a deterministic way set by the Gouy-phase coefficients. We therefore expect the HOSTP basis to serve as a versatile platform for designing and controlling genuinely four-dimensional (3D space and time) optical skyrmions and related topological textures.

Finally, the key features of spatiotemporal evolution and propagation dynamics revealed in HOSTP open new avenues for fundamental studies (toroidal electrodynamics, topological optics, and light–matter interaction) as well as for practical applications including precision metrology, particle acceleration, pulse compression, and optical communications.

## Supplementary Material

Supplementary Material Details
