# Supplementary material for: Higher-order spatiotemporal wave packets with Gouy phase dynamics
Source: Nanophotonics. 2025 Dec 10;14(24):4435–46. doi: 10.1515/nanoph-2025-0508 (PMC12704487; doi:10.1515/nanoph-2025-0508)
Supplement: Supplementary file 1 — Supplementary Material Details [file j_nanoph-2025-0508_suppl_001.pdf]

Supplementary Information A: Derivation of local-time formation of fundamental flying pancake pulse.

The TE-mode electric field of flying pancake pulse is given by:

$$E = 2f_0\sqrt{\frac{\mu_0}{\varepsilon_0}}\frac{(q_1+i\tau)^2-(q_2-i\sigma)^2}{\left[r^2+(q_1+i\tau)(q_2-i\sigma)\right]^3} \tag{A1}$$

Where $\tau = z-ct$, $\sigma = z+ct$, and $c = 1/\sqrt{\mu_0\varepsilon_0}$ is the speed of light. For the paraxial limit condition, $q_2 \gg q_1$, then $(q_2-i\sigma)^2 \gg (q_1+i\tau)^2$, the electric field can be simplified as:

$$E = -2f_0\sqrt{\frac{\mu_0}{\varepsilon_0}}\frac{(q_2-i\sigma)^2}{\left[r^2+(q_1+i\tau)(q_2-i\sigma)\right]^3} = -2f_0\sqrt{\frac{\mu_0}{\varepsilon_0}}\frac{1}{q_2-i\sigma}\cdot\frac{1}{\left[\dfrac{r^2}{q_2-i\sigma}+(q_1+i\tau)\right]^3} \tag{A2}$$

Considering the field is a very short propagating pulse at the speed of *c*, and $\tau = z-ct$ represents the local time, we can use the approximation of $z \doteq ct$to evaluate the $\sigma = z+ct \doteq 2z$, then the electric field can be derived as:

$$\begin{aligned}
E &= -2f_0\sqrt{\frac{\mu_0}{\varepsilon_0}}\frac{1}{q_2-i2z}\cdot\frac{1}{\left[q_1+\dfrac{r^2}{q_2-i2z}+i\tau\right]^3} \\
&= -2f_0\sqrt{\frac{\mu_0}{\varepsilon_0}}\frac{q_2+i2z}{4z^2+q_2^2}\cdot\frac{1}{\left[q_1+\dfrac{r^2(q_2+i2z)}{4z^2+q_2^2}+i\tau\right]^3} \\
&= -2f_0\sqrt{\frac{\mu_0}{\varepsilon_0}}\frac{q_2+i2z}{4z^2+q_2^2}\cdot\frac{1}{\left[q_1+\dfrac{q_2r^2}{4z^2+q_2^2}+i\left(\tau+\dfrac{2zr^2}{4z^2+q_2^2}\right)\right]^3}
\end{aligned} \tag{A3}$$

Here we define the notations of radius of curvature, *R*(*z*), and beam waist profile, *w*(*z*), as

$$R(z) = z\left[1+\left(\frac{z_0}{z}\right)^2\right] = \frac{4z^2+q_2^2}{4z} \tag{A4}$$

$$w^2(z) = w_0^2\left[1+\left(\frac{z}{z_0}\right)^2\right] = \frac{q_1}{2q_2}\left(4z^2+q_2^2\right) \tag{A5}$$

Where the Rayleigh length and basic beam waist constant are given by $z_0 = \frac{q_2}{2}$ and $w_0^2 = \frac{q_1q_2}{2} = q_1z_0$. Substitute these two notations and equations (A4) and (A5) into equation (A3), we can further simplify the electric field expression as:

$$E=-2f_0\sqrt{\frac{\mu_0}{\varepsilon_0}}\frac{q_2\left(1+i\frac{2z}{q_2}\right)}{\frac{2q_2}{q_1}w^2}\cdot\frac{1}{\left[q_1\left(1+\frac{r^2}{2w^2}\right)+i\left(\tau+\frac{r^2}{2R}\right)\right]^3}$$
$$=-f_0\sqrt{\frac{\mu_0}{\varepsilon_0}}\frac{w_0^2}{z_0w^2}\left(1+i\frac{z}{z_0}\right)\cdot\frac{1}{\left[q_1\left(1+\frac{r^2}{2w^2}\right)+i\left(\tau+\frac{r^2}{2R}\right)\right]^3} \quad \text{(A6)}$$

Applying Taylor expansion of $\sqrt{1+x^2}\exp\left(i\tan^{-1}x\right)=1+ix+O\left(\frac{x^2}{\sqrt{2}}\right)$, i.e. the approximation of

$$1+ix \doteq \sqrt{1+x^2}\,exp(i\,tan^{-1}x) \quad \text{(A7)}$$

the numerator term in equation (A6) can be rewritten as:

$$1+i\frac{z}{z_0}=\sqrt{1+\left(\frac{z}{z_0}\right)^2}\exp\left[i\cdot\tan^{-1}\left(\frac{z}{z_0}\right)\right]=\frac{w}{w_0}\exp\left[i\phi(z)\right] \quad \text{(A8)}$$

Where the $\phi(z)=\tan^{-1}\left(\frac{z}{z_0}\right)$ is the Gouy phase. Substitute equation (A8) into (A6) to carry on the simplification:

$$E=-f_0\sqrt{\frac{\mu_0}{\varepsilon_0}}\frac{w_0}{z_0w}\exp\left[i\phi(z)\right]\cdot\frac{1}{\left[q_1\left(1+\frac{r^2}{2w^2}\right)+i\left(\tau+\frac{r^2}{2R}\right)\right]^3}$$
$$=-f_0\sqrt{\frac{\mu_0}{\varepsilon_0}}\frac{w_0}{z_0w}\cdot\frac{\left[q_1\left(1+\frac{r^2}{2w^2}\right)-i\left(\tau+\frac{r^2}{2R}\right)\right]^3}{\left[\left(q_1\left(1+\frac{r^2}{2w^2}\right)\right)^2+\left(\tau+\frac{r^2}{2R}\right)^2\right]^3}\cdot\exp\left[i\phi(z)\right] \quad \text{(A9)}$$

Define the notations of radially scaled local time, $T(\mathbf{r},\tau)$, as

$$T=\frac{-\left(\tau+\frac{r^2}{2R}\right)}{q_1\left(1+\frac{r^2}{2w^2}\right)}=\frac{c\left(t-\frac{z+r^2/2R}{c}\right)}{q_1\left(1+\frac{r^2}{2w^2}\right)} \quad \text{(A10)}$$

Substitute equation (A10) into (A9) to carry on the simplification:

$$E = -f_0\sqrt{\frac{\mu_0}{\varepsilon_0}}\frac{w_0}{z_0 w}\cdot\frac{\left[q_1\left(1+\frac{r^2}{2w^2}\right)\right]^3 (1+iT)^3}{\left[\left(q_1\left(1+\frac{r^2}{2w^2}\right)\right)^2 \left(1+T^2\right)\right]^3}\cdot\exp\left[i\phi(z)\right]$$

$$= -f_0\sqrt{\frac{\mu_0}{\varepsilon_0}}\frac{w_0}{z_0 w}\cdot\frac{(1+iT)^3}{\left(q_1\left(1+\frac{r^2}{2w^2}\right)\right)^3 \left(1+T^2\right)^3}\cdot\exp\left[i\phi(z)\right] \tag{A11}$$

Applying the Taylor approximation of equation (A7) to simplify the numerator term in equation (A11), we get:

$$(1+iT)^3 \doteq \left[\sqrt{1+T^2}\, exp(i\, tan^{-1} T)\right]^3 = (1+T^2)^{3/2}\, exp(i\cdot 3\, tan^{-1} T) \tag{A12}$$

and carry on the simplification of the electric field expression:

$$E = -f_0\sqrt{\frac{\mu_0}{\varepsilon_0}}\frac{w_0}{z_0 w}\cdot\frac{\left(1+T^2\right)^{3/2}\exp\left(i\cdot 3\tan^{-1}T\right)}{\left(q_1\left(1+\frac{r^2}{2w^2}\right)\right)^3 \left(1+T^2\right)^3}\cdot\exp\left[i\phi(z)\right]$$

$$= -f_0\sqrt{\frac{\mu_0}{\varepsilon_0}}\frac{w_0}{z_0 w}\frac{1}{\left(q_1\left(1+\frac{r^2}{2w^2}\right)\right)^3 \left(1+T^2\right)^{3/2}}\cdot\exp\left\{i\left[3\tan^{-1}T+\phi(z)\right]\right\} \tag{A13}$$

Define the notations of local-time amplitude, $A(T)$, and local-time wavenumber, $k(T)$, as

$$A(T) = -f_0\sqrt{\frac{\mu_0}{\varepsilon_0}}\frac{1}{q_1^3 (T^2+1)^{3/2}} = \frac{-f_0\mu_0 c}{q_1^3 (T^2+1)^{3/2}} \tag{A14}$$

$$k(T) = 3\tan^{-1} T \tag{A15}$$

Substitute equations (A14) and (A15) into (A13), the final closed-form amplitude-phase expression is given by:

$$E = \frac{w_0 A(T)}{z_0 w\left(1+\frac{r^2}{2w^2}\right)^3}\exp\{i[k(T)+\phi(z)]\} \tag{A16}$$

Supplementary Information B: Derivation of local-time formation of fundamental flying doughnut pulse.

The TE-mode electric field of flying doughnut pulse is given by:

$$E = -4if_0\sqrt{\frac{\mu_0}{\varepsilon_0}}\frac{r(q_1+q_2-2ict)}{\left[r^2+(q_1+i\tau)(q_2-i\sigma)\right]^3} \tag{B1}$$

Where $\tau = z-ct$, $\sigma = z+ct$, and $c = 1/\sqrt{\mu_0\varepsilon_0}$ is the speed of light. For the paraxial limit condition, $q_2 \gg q_1$, the electric field can be simplified as:

$$E = -4if_0\sqrt{\frac{\mu_0}{\varepsilon_0}}\frac{r(q_2-2ict)}{\left[r^2+(q_1+i\tau)(q_2-i\sigma)\right]^3} \tag{B2}$$

Considering the field is a very short propagating pulse at the speed of c, and $\tau = z-ct$ represents the local time, we can use the approximation of $z \doteq ct$ to evaluate the $\sigma = z + ct \doteq 2z \doteq 2ct$, then the electric field can be derived as:

$$\begin{aligned}
E &= -4if_0\sqrt{\frac{\mu_0}{\varepsilon_0}}\frac{r(q_2-i2z)}{\left[r^2+(q_1+i\tau)(q_2-i2z)\right]^3} \\
&= -4if_0\sqrt{\frac{\mu_0}{\varepsilon_0}}\frac{r}{(q_2-i2z)^2}\cdot\frac{1}{\left[\dfrac{r^2}{q_2-i2z}+(q_1+i\tau)\right]^3} \\
&= -4if_0\sqrt{\frac{\mu_0}{\varepsilon_0}}r\left(\frac{q_2+i2z}{4z^2+q_2^2}\right)^2\cdot\frac{1}{\left[q_1+\dfrac{q_2r^2}{4z^2+q_2^2}+i\left(\tau+\dfrac{2zr^2}{4z^2+q_2^2}\right)\right]^3}
\end{aligned} \tag{B3}$$

Here we define the notations of radius of curvature, $R(z)$, and beam waist profile, $w(z)$, as equations (A4) and (A5), with the Rayleigh length and basic waist constant given by $z_0 = \frac{q_2}{2}$ and $w_0^2 = \frac{q_1q_2}{2} = q_1z_0$. Substitute these two notations and equations (A4) and (A5) into equation (B3), we can simplify electric field expression as:

$$\begin{aligned}
E &= -4if_0\sqrt{\frac{\mu_0}{\varepsilon_0}}r\left[\frac{q_2\left(1+i\dfrac{2z}{q_2}\right)}{\dfrac{2q_2}{q_1}w^2}\right]^2\cdot\frac{1}{\left[q_1\left(1+\dfrac{r^2}{2w^2}\right)+i\left(\tau+\dfrac{r^2}{2R}\right)\right]^3} \\
&= -4if_0\sqrt{\frac{\mu_0}{\varepsilon_0}}r\left[\frac{w_0^2\left(1+i\dfrac{z}{z_0}\right)}{2z_0w^2}\right]^2\cdot\frac{1}{\left[q_1\left(1+\dfrac{r^2}{2w^2}\right)+i\left(\tau+\dfrac{r^2}{2R}\right)\right]^3} \\
&= -if_0\sqrt{\frac{\mu_0}{\varepsilon_0}}r\frac{w_0^4}{z_0^2w^4}\left(1+i\frac{z}{z_0}\right)^2\cdot\frac{1}{\left[q_1\left(1+\dfrac{r^2}{2w^2}\right)+i\left(\tau+\dfrac{r^2}{2R}\right)\right]^3}
\end{aligned} \tag{B4}$$

Applying Taylor approximation of equation (A7), the numerator term in equation (B4) can be rewritten as:

$$\left(1+i\frac{z}{z_0}\right)^2=\left\{\sqrt{1+\left(\frac{z}{z_0}\right)^2}\exp\left[i\cdot\tan^{-1}\left(\frac{z}{z_0}\right)\right]\right\}^2=\frac{w^2}{w_0^2}\exp\left[i2\phi(z)\right] \tag{B5}$$

Where the $\phi(z)=\tan^{-1}\left(\frac{z}{z_0}\right)$ is the Gouy phase. Substitute equation (B5) into (B4) to carry on the simplification:

$$\begin{aligned}E&=-if_0\sqrt{\frac{\mu_0}{\varepsilon_0}}r\frac{w_0^2}{z_0^2w^2}\exp\left[i2\phi(z)\right]\cdot\frac{1}{\left[q_1\left(1+\frac{r^2}{2w^2}\right)+i\left(\tau+\frac{r^2}{2R}\right)\right]^3}\\&=-if_0\sqrt{\frac{\mu_0}{\varepsilon_0}}\frac{rw_0^2}{z_0^2w^2}\cdot\frac{\left[q_1\left(1+\frac{r^2}{2w^2}\right)-i\left(\tau+\frac{r^2}{2R}\right)\right]^3}{\left[\left(q_1\left(1+\frac{r^2}{2w^2}\right)\right)^2+\left(\tau+\frac{r^2}{2R}\right)^2\right]^3}\cdot\exp\left[i2\phi(z)\right]\end{aligned} \tag{B6}$$

Define the notations of radially scaled local time, $T(\mathbf{r},\tau)$, as equation (A10), and substitute equation (A10) into (B6) to carry on the simplification:

$$\begin{aligned}E&=-if_0\sqrt{\frac{\mu_0}{\varepsilon_0}}\frac{rw_0^2}{z_0^2w^2}\cdot\frac{\left[q_1\left(1+\frac{r^2}{2w^2}\right)\right]^3(1+iT)^3}{\left[\left(q_1\left(1+\frac{r^2}{2w^2}\right)\right)^2\left(1+T^2\right)\right]^3}\cdot\exp\left[i2\phi(z)\right]\\&=-if_0\sqrt{\frac{\mu_0}{\varepsilon_0}}\frac{rw_0^2}{z_0^2w^2}\cdot\frac{(1+iT)^3}{\left(q_1\left(1+\frac{r^2}{2w^2}\right)\right)^3\left(1+T^2\right)^3}\cdot\exp\left[i2\phi(z)\right]\end{aligned} \tag{B7}$$

Applying the Taylor approximation of equation (A12) to simplify the numerator term in equation (B7), we get:

$$\begin{aligned}E&=-if_0\sqrt{\frac{\mu_0}{\varepsilon_0}}\frac{rw_0^2}{z_0^2w^2}\cdot\frac{\left(1+T^2\right)^{3/2}\exp\left(i\cdot3\tan^{-1}T\right)}{\left(q_1\left(1+\frac{r^2}{2w^2}\right)\right)^3\left(1+T^2\right)^3}\cdot\exp\left[i\phi(z)\right]\\&=-if_0\sqrt{\frac{\mu_0}{\varepsilon_0}}\frac{rw_0^2}{z_0^2w^2}\frac{1}{\left(q_1\left(1+\frac{r^2}{2w^2}\right)\right)^3\left(1+T^2\right)^{3/2}}\cdot\exp\left\{i\left[3\tan^{-1}T+2\phi(z)\right]\right\}\end{aligned} \tag{B8}$$

Define the notations of local-time amplitude, $A(T)$, and local-time wavenumber, $k(T)$, as equations (A14) and (A16), substitute equations (A14) and (A15) into (B8), the final closed-form amplitude-phase expression is given by:

$$E=i\frac{w_0^2rA(T)}{z_0^2w^2\left(1+\frac{r^2}{2w^2}\right)^3}\exp\left\{i[k(T)+2\phi(z)]\right\} \tag{B9}$$

Supplementary Information C: Derivation of local-time formation of high-order flying pancake pulse.

We start from the scalar generating function in EDEPT method:

$$f = f_0 \frac{e^{-s/q_3}}{(q_1 + i\tau)(s + q_2)^{\alpha}} \tag{C1}$$

Where $s \equiv r^2/(q_1 + i\tau) - i\sigma$, $\tau = z - ct$, $\sigma = z + ct$, $r = \sqrt{x^2 + y^2}$, $c = 1/\sqrt{\mu_0 \varepsilon_0}$ is the speed of light, the parameters q_1, q_2, q_3 are real positive with units of length, and the real dimensionless parameter α must satisfy $\alpha \geq 1$ in order for the electromagnetic pulse to fulfill finite energy. In conventional method, it always assumes that $q_3 \to \infty$ and $\alpha = 1$. Here we break the limit of the parameter α, that can be any real number no less than one in our new derivation. The scalar generating function is given by:

$$\begin{aligned} f &= f_0 \frac{1}{(q_1 + i\tau)\left(\frac{r^2}{q_1 + i\tau} - i\sigma + q_2\right)^{\alpha}} \\ &= f_0 \frac{(q_1 + i\tau)^{\alpha - 1}}{\left[r^2 + (q_1 + i\tau)(q_2 - i\sigma)\right]^{\alpha}} \end{aligned} \tag{C2}$$

The pancake-like pulse is derived under a linear vector Hertz potential $\mathbf{\Pi} = \hat{\mathbf{x}} f(\mathbf{r}, t)$ in Cartesian coordinate (x, y, z), the TE-mode electromagnetic field can be generated from Hertz potential by

$$\begin{cases} \mathbf{E}(\mathbf{r}, t) = -\mu_0 \dfrac{\partial}{\partial t} \nabla \times \mathbf{\Pi} = \mu_0 \left(-\hat{\mathbf{y}} \partial_z \partial_t f + \hat{\mathbf{z}} \partial_y \partial_t f\right) \\ \mathbf{H}(\mathbf{r}, t) = \nabla \times (\nabla \times \mathbf{\Pi}) = \hat{\mathbf{x}} \left(\partial_z^2 - \dfrac{1}{c^2} \partial_t^2\right) f + \hat{\mathbf{y}} \partial_x \partial_y f + \hat{\mathbf{z}} \partial_z \partial_x f \end{cases} \tag{C3}$$

For the paraxial pulse solution, the electric field has linear polarization (at transverse direction e.g. y-direction) and its z-component can be neglected, which can be derived as:

$$\begin{aligned} E &= -\mu_0 \partial_z \partial_t f = -\mu_0 f_0 \partial_z \partial_t \frac{(q_1 + i\tau)^{\alpha - 1}}{\left[r^2 + (q_1 + i\tau)(q_2 - i\sigma)\right]^{\alpha}} \\ &= \mu_0 f_0 \left\{ \frac{\alpha(\alpha + 1) c (q_1 + i\tau)^{\alpha - 1} \left[(q_1 + i\tau)^2 - (q_2 - i\sigma)^2\right]}{\left[r^2 + (q_1 + i\tau)(q_2 - i\sigma)\right]^{\alpha + 2}} + \frac{2(\alpha - 1)\alpha c (q_1 + i\tau)^{\alpha - 2} (q_2 - i\sigma)}{\left[r^2 + (q_1 + i\tau)(q_2 - i\sigma)\right]^{\alpha + 1}} - \frac{(\alpha - 2)(\alpha - 1) c (q_1 + i\tau)^{\alpha - 3}}{\left[r^2 + (q_1 + i\tau)(q_2 - i\sigma)\right]^{\alpha}} \right\} \\ &= f_0 \sqrt{\frac{\mu_0}{\varepsilon_0}} \left\{ \frac{\alpha(\alpha + 1)(q_1 + i\tau)^{\alpha - 1} \left[(q_1 + i\tau)^2 - (q_2 - i\sigma)^2\right]}{\left[r^2 + (q_1 + i\tau)(q_2 - i\sigma)\right]^{\alpha + 2}} + \frac{2(\alpha - 1)\alpha (q_1 + i\tau)^{\alpha - 2} (q_2 - i\sigma)}{\left[r^2 + (q_1 + i\tau)(q_2 - i\sigma)\right]^{\alpha + 1}} - \frac{(\alpha - 2)(\alpha - 1)(q_1 + i\tau)^{\alpha - 3}}{\left[r^2 + (q_1 + i\tau)(q_2 - i\sigma)\right]^{\alpha}} \right\} \end{aligned}$$

(C4)

This equation (C4) is the expression of high-order flying pancake pulse. Note that when $\alpha = 1$, the equation (C4) will be reduced into the expression of fundamental flying pancake pulse as equation (A1). The second and third terms in the curly bracket of equation (C4) are extremely small value, that can be neglected in calculation because the denominator is extremely larger than the numerator for both terms. For the paraxial limit condition, $q_2 \gg q_1$, there is $(q_2 - i\sigma)^2 \gg (q_1 + i\tau)^2$ in the first term in the curly bracket of equation (C4), the electric field can be simplified as:

$$E = f_0\sqrt{\frac{\mu_0}{\varepsilon_0}}\frac{\alpha(\alpha+1)(q_1+i\tau)^{\alpha-1}\left[(q_1+i\tau)^2-(q_2-i\sigma)^2\right]}{\left[r^2+(q_1+i\tau)(q_2-i\sigma)\right]^{\alpha+2}}$$
$$= -\alpha(\alpha+1)f_0\sqrt{\frac{\mu_0}{\varepsilon_0}}\frac{(q_1+i\tau)^{\alpha-1}(q_2-i\sigma)^2}{\left[r^2+(q_1+i\tau)(q_2-i\sigma)\right]^{\alpha+2}} \tag{C5}$$
$$= -\alpha(\alpha+1)f_0\sqrt{\frac{\mu_0}{\varepsilon_0}}(q_1+i\tau)^{\alpha-1}\frac{1}{(q_2-i\sigma)^{\alpha}}\frac{1}{\left[\frac{r^2}{q_2-i\sigma}+(q_1+i\tau)\right]^{\alpha+2}}$$

Considering the field is a very short propagating pulse at the speed of c, and $\tau = z - ct$ represents the local time, we can use the approximation of $z \doteq ct$to evaluate the $\sigma = z + ct \doteq 2z$, then the electric field can be derived as:

$$E = -\alpha(\alpha+1)f_0\sqrt{\frac{\mu_0}{\varepsilon_0}}(q_1+i\tau)^{\alpha-1}\frac{1}{(q_2-i2z)^{\alpha}}\frac{1}{\left[q_1+\frac{r^2}{q_2-i2z}+i\tau\right]^{\alpha+2}}$$
$$= -\alpha(\alpha+1)f_0\sqrt{\frac{\mu_0}{\varepsilon_0}}(q_1+i\tau)^{\alpha-1}\left(\frac{q_2+i2z}{4z^2+q_2^2}\right)^{\alpha}\frac{1}{\left[q_1+\frac{r^2(q_2+i2z)}{4z^2+q_2^2}+i\tau\right]^{\alpha+2}} \tag{C6}$$
$$= -\alpha(\alpha+1)f_0\sqrt{\frac{\mu_0}{\varepsilon_0}}(q_1+i\tau)^{\alpha-1}\left(\frac{q_2+i2z}{4z^2+q_2^2}\right)^{\alpha}\frac{1}{\left[q_1+\frac{q_2r^2}{4z^2+q_2^2}+i\left(\tau+\frac{2zr^2}{4z^2+q_2^2}\right)\right]^{\alpha+2}}$$

Here we define the notations of radius of curvature, $R(z)$, and beam waist profile, $w(z)$, as equations (A4) and (A5), with the Rayleigh length and basic waist constant given by $z_0 = \frac{q_2}{2}$ and $w_0^2 = \frac{q_1q_2}{2} = q_1z_0$. Substitute these two notations and equations (A4) and (A5) into equation (C6), we can simplify electric field expression as:

$$E = -\alpha(\alpha+1)f_0\sqrt{\frac{\mu_0}{\varepsilon_0}}(q_1+i\tau)^{\alpha-1}\left[\frac{q_2\left(1+i\frac{2z}{q_2}\right)}{\frac{2q_2}{q_1}w^2}\right]^{\alpha}\frac{1}{\left[q_1\left(1+\frac{r^2}{2w^2}\right)+i\left(\tau+\frac{r^2}{2R}\right)\right]^{\alpha+2}}$$
$$= -\alpha(\alpha+1)f_0\sqrt{\frac{\mu_0}{\varepsilon_0}}(q_1+i\tau)^{\alpha-1}\left[\frac{w_0^2\left(1+i\frac{z}{z_0}\right)}{2z_0w^2}\right]^{\alpha}\frac{1}{\left[q_1\left(1+\frac{r^2}{2w^2}\right)+i\left(\tau+\frac{r^2}{2R}\right)\right]^{\alpha+2}} \tag{C7}$$
$$= -\frac{\alpha(\alpha+1)}{2^{\alpha}}f_0\sqrt{\frac{\mu_0}{\varepsilon_0}}(q_1+i\tau)^{\alpha-1}\frac{w_0^{2\alpha}}{z_0^{\alpha}w^{2\alpha}}\left(1+i\frac{z}{z_0}\right)^{\alpha}\frac{1}{\left[q_1\left(1+\frac{r^2}{2w^2}\right)+i\left(\tau+\frac{r^2}{2R}\right)\right]^{\alpha+2}}$$

Applying Taylor approximation of equation (A7), the numerator term in equation (C7) can be rewritten as:

$$\left(1+i\frac{z}{z_0}\right)^{\alpha} = \left\{\sqrt{1+\left(\frac{z}{z_0}\right)^2}\exp\left[i\cdot\tan^{-1}\left(\frac{z}{z_0}\right)\right]\right\}^{\alpha} = \frac{w^{\alpha}}{w_0^{\alpha}}\exp\left[i\alpha\phi(z)\right] \tag{C8}$$

Where the $\phi(z)=\tan^{-1}\left(\frac{z}{z_0}\right)$ is the Gouy phase. Substitute equation (C8) into (C7) to carry on the simplification:

$$\begin{aligned} E &= -\frac{\alpha(\alpha+1)}{2^{\alpha}} f_0\sqrt{\frac{\mu_0}{\varepsilon_0}}(q_1+i\tau)^{\alpha-1}\frac{w_0^{\alpha}}{z_0^{\alpha}w^{\alpha}}\exp\left[i\alpha\phi(z)\right]\cdot\frac{1}{\left[q_1\left(1+\frac{r^2}{2w^2}\right)+i\left(\tau+\frac{r^2}{2R}\right)\right]^{\alpha+2}} \\ &= -\frac{\alpha(\alpha+1)}{2^{\alpha}} f_0\sqrt{\frac{\mu_0}{\varepsilon_0}}\frac{w_0^{\alpha}(q_1+i\tau)^{\alpha-1}}{z_0^{\alpha}w^{\alpha}}\cdot\frac{\left[q_1\left(1+\frac{r^2}{2w^2}\right)-i\left(\tau+\frac{r^2}{2R}\right)\right]^{\alpha+2}}{\left[\left(q_1\left(1+\frac{r^2}{2w^2}\right)\right)^2+\left(\tau+\frac{r^2}{2R}\right)^2\right]^{\alpha+2}}\cdot\exp\left[i\alpha\phi(z)\right] \end{aligned} \tag{C9}$$

Define the notations of radially scaled local time, $T(\mathbf{r},\tau)$, as equation (A10), and substitute equation (A10) into (C9) to carry on the simplification:

$$\begin{aligned} E &= -\frac{\alpha(\alpha+1)}{2^{\alpha}} f_0\sqrt{\frac{\mu_0}{\varepsilon_0}}\frac{w_0^{\alpha}(q_1+i\tau)^{\alpha-1}}{z_0^{\alpha}w^{\alpha}}\cdot\frac{\left[q_1\left(1+\frac{r^2}{2w^2}\right)\right]^{\alpha+2}\left(1+iT\right)^{\alpha+2}}{\left[\left(q_1\left(1+\frac{r^2}{2w^2}\right)\right)^2\left(1+T^2\right)\right]^{\alpha+2}}\cdot\exp\left[i\alpha\phi(z)\right] \\ &= -\frac{\alpha(\alpha+1)}{2^{\alpha}} f_0\sqrt{\frac{\mu_0}{\varepsilon_0}}\frac{w_0^{\alpha}(q_1+i\tau)^{\alpha-1}}{z_0^{\alpha}w^{\alpha}}\cdot\frac{\left(1+iT\right)^{\alpha+2}}{\left(q_1\left(1+\frac{r^2}{2w^2}\right)\right)^{\alpha+2}\left(1+T^2\right)^{\alpha+2}}\cdot\exp\left[i\alpha\phi(z)\right] \end{aligned} \tag{C10}$$

Applying the Taylor approximation of equation (A7) to simplify the numerator term in equation (C10), we get:

$$(1+iT)^{\alpha+2} \doteq \left[\sqrt{1+T^2}\,exp(i\,tan^{-1}\,T)\right]^{\alpha+2} = (1+T^2)^{(\alpha+2)/2}\,exp[i\cdot(\alpha+2)\,tan^{-1}\,T] \tag{C11}$$

and carry on the simplification of the electric field expression:

$$\begin{aligned} E &= -\frac{\alpha(\alpha+1)}{2^{\alpha}} f_0\sqrt{\frac{\mu_0}{\varepsilon_0}}\frac{w_0^{\alpha}(q_1+i\tau)^{\alpha-1}}{z_0^{\alpha}w^{\alpha}}\cdot\frac{\left(1+T^2\right)^{(\alpha+2)/2}\exp\left[i\cdot(\alpha+2)\tan^{-1}T\right]}{\left(q_1\left(1+\frac{r^2}{2w^2}\right)\right)^{\alpha+2}\left(1+T^2\right)^{\alpha+2}}\cdot\exp\left[i\alpha\phi(z)\right] \\ &= -\frac{\alpha(\alpha+1)}{2^{\alpha}} f_0\sqrt{\frac{\mu_0}{\varepsilon_0}}\frac{w_0^{\alpha}(q_1+i\tau)^{\alpha-1}}{z_0^{\alpha}w^{\alpha}\left(q_1\left(1+\frac{r^2}{2w^2}\right)\right)^{\alpha+2}\left(1+T^2\right)^{(\alpha+2)/2}}\cdot\exp\left\{i\left[(\alpha+2)\tan^{-1}T+\alpha\phi(z)\right]\right\} \end{aligned} \tag{C12}$$

The numerator term in equation (C12) can be further simplified by using the Taylor approximation of equation (A7) to separate the amplitude and phase terms as:

$$(q_1+i\tau)^{\alpha-1}=\left[q_1\left(1+i\frac{\tau}{q_1}\right)\right]^{\alpha-1}=\left(q_1\sqrt{1+\left(\frac{\tau}{q_1}\right)^2}\exp\left[i\tan^{-1}\left(\frac{\tau}{q_1}\right)\right]\right)^{\alpha-1}$$
$$=\left(q_1^2+\tau^2\right)^{(\alpha-1)/2}\exp\left[i(\alpha-1)\tan^{-1}\left(\frac{\tau}{q_1}\right)\right] \tag{C13}$$

Substitute equation (C13) into (C12) to carry on the derivation:

$$E=-\frac{\alpha(\alpha+1)}{2^\alpha}f_0\sqrt{\frac{\mu_0}{\varepsilon_0}}\frac{w_0^\alpha\left(q_1^2+\tau^2\right)^{(\alpha-1)/2}}{z_0^\alpha w^\alpha\left(q_1\left(1+\frac{r^2}{2w^2}\right)\right)^{\alpha+2}\left(1+T^2\right)^{(\alpha+2)/2}}\cdot\exp\left\{i\left[(\alpha-1)\tan^{-1}\left(\frac{\tau}{q_1}\right)+(\alpha+2)\tan^{-1}T+\alpha\phi(z)\right]\right\} \tag{C14}$$

Define the notations of generalized local-time amplitude, $A_\alpha(\mathbf{r},\tau)$, and generalized local-time wavenumber, $k_\alpha(\mathbf{r},\tau)$, as

$$A_\alpha(\mathbf{r},\tau)=-f_0\sqrt{\frac{\mu_0}{\varepsilon_0}}\frac{\left(q_1^2+\tau^2\right)^{(\alpha-1)/2}}{q_1^{\alpha+2}(T^2+1)^{(\alpha+2)/2}}=\frac{-f_0\mu_0 c\left(q_1^2+\tau^2\right)^{(\alpha-1)/2}}{q_1^{\alpha+2}(T^2+1)^{(\alpha+2)/2}} \tag{C15}$$

$$k_\alpha(\mathbf{r},\tau)=(\alpha-1)\tan^{-1}\left(\frac{\tau}{q_1}\right)+(\alpha+2)\tan^{-1}T \tag{C16}$$

Note that when $\alpha=1$, the generalized local-time amplitude, $A_\alpha(\mathbf{r},\tau)$, and generalized local-time wavevector, $k_\alpha(\mathbf{r},\tau)$, equations (C15) and (C16) will be reduced into the fundamental local-time amplitude, $A(T)$, and fundamental local-time wavevector, $k(T)$, equations (A14) and (A15). Substitute equations (C15) and (C16) into (C14), the final closed-form amplitude-phase expression is given by:

$$E=\frac{\alpha(\alpha+1)w_0^\alpha A_\alpha(\mathbf{r},\tau)}{2^\alpha z_0^\alpha w^\alpha\left(1+\frac{r^2}{2w^2}\right)^{\alpha+2}}\exp\{i[k_\alpha(\mathbf{r},\tau)+\alpha\phi(z)]\} \tag{C17}$$

Note that when $\alpha=1$, the amplitude-phase expression of high-order flying pancake pulse of equation (C17) will be reduced into the amplitude-phase expression of fundamental flying pancake pulse as equation (A16). Also, when the index of α goes higher, the energy of the pulse would be smaller because $\frac{\alpha(\alpha+1)}{2^\alpha}\to 0$ when α goes to infinity, that is consistent with the meaning in the finite-energy assumption in the EDEPT method.

Supplementary Information D: Derivation of local-time formation of high-order flying doughnut pulse.

Based on the scalar generating function in EDEPT method as equation (C2), the doughnut-like pulse is derived under a curled vector Hertz potential $\boldsymbol{\Pi} = \hat{\mathbf{z}} f(\mathbf{r}, t)$ in cylindrical coordinate (r, θ, z), the TE-mode electromagnetic field can be generated from Hertz potential by

$$\begin{cases} \mathbf{E}(\mathbf{r},t) = -\mu_0 \dfrac{\partial}{\partial t} \nabla \times \mathbf{\Pi} = \hat{\boldsymbol{\theta}} \mu_0 \partial_r \partial_t f \\ \mathbf{H}(\mathbf{r},t) = \nabla \times \left(\nabla \times \mathbf{\Pi} \right) = \hat{\mathbf{r}} \partial_r \partial_z f + \hat{\mathbf{z}} \left(\partial_z^2 - \dfrac{1}{c^2} \partial_t^2 \right) f \end{cases} \tag{D1}$$

For this pulse solution, the electric field is purely azimuthally polarized, and the magnetic field is along the radial and longitudinal directions with no azimuthal component. The azimuthally polarized electric field is derived as:

$$\begin{aligned} E &= \mu_0 \partial_z \partial_t f = \mu_0 f_0 \partial_\rho \partial_t \frac{\left(q_1 + i\tau\right)^{\alpha-1}}{\left[r^2 + \left(q_1 + i\tau\right)\left(q_2 - i\sigma\right)\right]^{\alpha}} \\ &= \mu_0 f_0 \left\{ \frac{-2\alpha(\alpha+1) icr (q_1 + i\tau)^{\alpha-1} (q_1 + q_2 - 2ict)}{\left[r^2 + \left(q_1 + i\tau\right)\left(q_2 - i\sigma\right)\right]^{\alpha+2}} + \frac{2(\alpha-1)\alpha icr (q_1 + i\tau)^{\alpha-2}}{\left[r^2 + \left(q_1 + i\tau\right)\left(q_2 - i\sigma\right)\right]^{\alpha+1}} \right\} \\ &= 2 f_0 \sqrt{\frac{\mu_0}{\varepsilon_0}} \left\{ \frac{-\alpha(\alpha+1) ir (q_1 + i\tau)^{\alpha-1} (q_1 + q_2 - 2ict)}{\left[r^2 + \left(q_1 + i\tau\right)\left(q_2 - i\sigma\right)\right]^{\alpha+2}} + \frac{(\alpha-1)\alpha ir (q_1 + i\tau)^{\alpha-2}}{\left[r^2 + \left(q_1 + i\tau\right)\left(q_2 - i\sigma\right)\right]^{\alpha+1}} \right\} \end{aligned} \tag{D2}$$

This equation (D2) is the expression of high-order flying doughnut pulse. Note that when $\alpha = 1$, the equation (D2) will be reduced into the expression of fundamental flying doughnut pulse as equation (B1). For the paraxial limit condition, $q_2 \gg q_1$, and neglecting extremely small value of the second term, the equation (D2) can be simplified as:

$$E = -2\alpha(\alpha+1) i f_0 \sqrt{\frac{\mu_0}{\varepsilon_0}} \frac{r (q_1 + i\tau)^{\alpha-1} (q_2 - 2ict)}{\left[r^2 + \left(q_1 + i\tau\right)\left(q_2 - i\sigma\right)\right]^{\alpha+2}} \tag{D3}$$

Considering the field is a very short propagating pulse at the speed of c, and $\tau = z - ct$ represents the local time, we can use the approximation of $z \doteq ct$to evaluate the $\sigma = z + ct \doteq 2z$, then the electric field can be derived as:

$$\begin{aligned} E &= -2\alpha(\alpha+1) i f_0 \sqrt{\frac{\mu_0}{\varepsilon_0}} \frac{r (q_1 + i\tau)^{\alpha-1} (q_2 - i2z)}{\left[r^2 + \left(q_1 + i\tau\right)\left(q_2 - i2z\right)\right]^{\alpha+2}} \\ &= -2\alpha(\alpha+1) i f_0 \sqrt{\frac{\mu_0}{\varepsilon_0}} r (q_1 + i\tau)^{\alpha-1} \frac{1}{\left(q_2 - i2z\right)^{\alpha+1}} \frac{1}{\left[\dfrac{r^2}{q_2 - i2z} + \left(q_1 + i\tau\right)\right]^{\alpha+2}} \\ &= -2\alpha(\alpha+1) i f_0 \sqrt{\frac{\mu_0}{\varepsilon_0}} r (q_1 + i\tau)^{\alpha-1} \left(\frac{q_2 + i2z}{4z^2 + q_2^2} \right)^{\alpha+1} \frac{1}{\left[q_1 + \dfrac{r^2 (q_2 + i2z)}{4z^2 + q_2^2} + i\tau\right]^{\alpha+2}} \\ &= -2\alpha(\alpha+1) i f_0 \sqrt{\frac{\mu_0}{\varepsilon_0}} r (q_1 + i\tau)^{\alpha-1} \left(\frac{q_2 + i2z}{4z^2 + q_2^2} \right)^{\alpha+1} \frac{1}{\left[q_1 + \dfrac{q_2 r^2}{4z^2 + q_2^2} + i\left(\tau + \dfrac{2zr^2}{4z^2 + q_2^2}\right)\right]^{\alpha+2}} \end{aligned} \tag{D4}$$

Here we define the notations of radius of curvature, $R(z)$, and beam waist profile, $w(z)$, as equations (A4) and (A5), with the Rayleigh length and basic waist constant given by $z_0 = \frac{q_2}{2}$ and $w_0^2 = \frac{q_1 q_2}{2} = q_1 z_0$. Substitute these two notations and equations (A4) and (A5) into equation (D4), we can simplify electric field expression as:

$$
\begin{aligned}
E &= -2\alpha(\alpha+1) i f_0 \sqrt{\frac{\mu_0}{\varepsilon_0}} r (q_1 + i\tau)^{\alpha-1} \left[\frac{q_2\left(1 + i\frac{2z}{q_2}\right)}{\frac{2q_2}{q_1} w^2} \right]^{\alpha+1} \frac{1}{\left[q_1\left(1 + \frac{r^2}{2w^2}\right) + i\left(\tau + \frac{r^2}{2R}\right) \right]^{\alpha+2}} \\
&= -2\alpha(\alpha+1) i f_0 \sqrt{\frac{\mu_0}{\varepsilon_0}} r (q_1 + i\tau)^{\alpha-1} \left[\frac{w_0^2\left(1 + i\frac{z}{z_0}\right)}{2z_0 w^2} \right]^{\alpha+1} \frac{1}{\left[q_1\left(1 + \frac{r^2}{2w^2}\right) + i\left(\tau + \frac{r^2}{2R}\right) \right]^{\alpha+2}} \qquad \text{(D5)} \\
&= -\frac{\alpha(\alpha+1)}{2^\alpha} i f_0 \sqrt{\frac{\mu_0}{\varepsilon_0}} r (q_1 + i\tau)^{\alpha-1} \frac{w_0^{2(\alpha+1)}}{z_0^{\alpha+1} w^{2(\alpha+1)}} \left(1 + i\frac{z}{z_0}\right)^{\alpha+1} \frac{1}{\left[q_1\left(1 + \frac{r^2}{2w^2}\right) + i\left(\tau + \frac{r^2}{2R}\right) \right]^{\alpha+2}}
\end{aligned}
$$

Applying Taylor approximation of equation (A7), the numerator term in equation (D5) can be rewritten as:

$$
\left(1 + i\frac{z}{z_0}\right)^{\alpha+1} = \left\{ \sqrt{1 + \left(\frac{z}{z_0}\right)^2} \exp\left[i \cdot \tan^{-1}\left(\frac{z}{z_0}\right) \right] \right\}^{\alpha+1} = \frac{w^{\alpha+1}}{w_0^{\alpha+1}} \exp\left[i(\alpha+1)\phi(z) \right] \qquad \text{(D6)}
$$

Where the $\phi(z) = \tan^{-1}\left(\frac{z}{z_0}\right)$ is the Gouy phase. Substitute equation (D6) into (D5) to carry on the simplification:

$$
\begin{aligned}
E &= -\frac{\alpha(\alpha+1)}{2^\alpha} i f_0 \sqrt{\frac{\mu_0}{\varepsilon_0}} r (q_1 + i\tau)^{\alpha-1} \frac{w_0^{2(\alpha+1)}}{z_0^{\alpha+1} w^{2(\alpha+1)}} \exp\left[i(\alpha+1)\phi(z) \right] \cdot \frac{1}{\left[q_1\left(1 + \frac{r^2}{2w^2}\right) + i\left(\tau + \frac{r^2}{2R}\right) \right]^{\alpha+2}} \\
&= -\frac{\alpha(\alpha+1)}{2^\alpha} i f_0 \sqrt{\frac{\mu_0}{\varepsilon_0}} \frac{w_0^{\alpha+1} r (q_1 + i\tau)^{\alpha-1}}{z_0^{\alpha+1} w^{\alpha+1}} \cdot \frac{\left[q_1\left(1 + \frac{r^2}{2w^2}\right) - i\left(\tau + \frac{r^2}{2R}\right) \right]^{\alpha+2}}{\left[\left(q_1\left(1 + \frac{r^2}{2w^2}\right)\right)^2 + \left(\tau + \frac{r^2}{2R}\right)^2 \right]^{\alpha+2}} \cdot \exp\left[i(\alpha+1)\phi(z) \right] \qquad \text{(D7)}
\end{aligned}
$$

Define the notations of radially scaled local time, $T(\mathbf{r},\tau)$, as equation (A10), and substitute equation (A10) into (D7) to carry on the simplification:

$$E = -\alpha(\alpha+1) i f_0 \sqrt{\frac{\mu_0}{\varepsilon_0}} \frac{r(q_1+i\tau)^{\alpha-1}}{2^\alpha \left(z^2+z_0^2\right)^{(\alpha+1)/2}} \cdot \frac{\left[q_1\left(1+\frac{r^2}{2w^2}\right)\right]^{\alpha+2} \left(1+iT\right)^{\alpha+2}}{\left[\left(q_1\left(1+\frac{r^2}{2w^2}\right)\right)^2 \left(1+T^2\right)\right]^{\alpha+2}} \cdot \exp\left[i(\alpha+1)\phi(z)\right]$$

$$= -\frac{\alpha(\alpha+1)}{2^\alpha} i f_0 \sqrt{\frac{\mu_0}{\varepsilon_0}} \frac{w_0^{\alpha+1} r(q_1+i\tau)^{\alpha-1}}{z_0^{\alpha+1} w^{\alpha+1}} \cdot \frac{\left(1+iT\right)^{\alpha+2}}{\left(q_1\left(1+\frac{r^2}{2w^2}\right)\right)^{\alpha+2} \left(1+T^2\right)^{\alpha+2}} \cdot \exp\left[i(\alpha+1)\phi(z)\right] \quad \text{(D8)}$$

Applying the Taylor approximation of equation (C11) to simplify the numerator term in equation (D8), we carry on the simplification of the electric field expression:

$$E = -\frac{\alpha(\alpha+1)}{2^\alpha} i f_0 \sqrt{\frac{\mu_0}{\varepsilon_0}} \frac{w_0^{\alpha+1} r(q_1+i\tau)^{\alpha-1}}{z_0^{\alpha+1} w^{\alpha+1}} \cdot \frac{\left(1+T^2\right)^{(\alpha+2)/2} \exp\left[i\cdot(\alpha+2)\tan^{-1}T\right]}{\left(q_1\left(1+\frac{r^2}{2w^2}\right)\right)^{\alpha+2} \left(1+T^2\right)^{\alpha+2}} \cdot \exp\left[i(\alpha+1)\phi(z)\right]$$

$$= -\frac{\alpha(\alpha+1)}{2^\alpha} i f_0 \sqrt{\frac{\mu_0}{\varepsilon_0}} \frac{w_0^{\alpha+1} r(q_1+i\tau)^{\alpha-1}}{z_0^{\alpha+1} w^{\alpha+1} \left(q_1\left(1+\frac{r^2}{2w^2}\right)\right)^{\alpha+2} \left(1+T^2\right)^{(\alpha+2)/2}} \cdot \exp\left\{i\left[(\alpha+2)\tan^{-1}T + (\alpha+1)\phi(z)\right]\right\} \quad \text{(D9)}$$

The numerator term in equation (D9) can be further simplified by using the Taylor approximation of equation (A7) to separate the amplitude and phase terms as equation (C13), and we substitute equation (C13) into (D9) to further derive it as:

$$E = -\frac{\alpha(\alpha+1)}{2^\alpha} i f_0 \sqrt{\frac{\mu_0}{\varepsilon_0}} \frac{w_0^{\alpha+1} r\left(q_1^2+\tau^2\right)^{(\alpha-1)/2}}{z_0^{\alpha+1} w^{\alpha+1} \left(q_1\left(1+\frac{r^2}{2w^2}\right)\right)^{\alpha+2} \left(1+T^2\right)^{(\alpha+2)/2}} \cdot \exp\left\{i\left[(\alpha-1)\tan^{-1}\left(\frac{\tau}{q_1}\right) + (\alpha+2)\tan^{-1}T + (\alpha+1)\phi(z)\right]\right\}$$

$$\text{(D10)}$$

Define the notations of generalized local-time amplitude, $A_\alpha(\mathbf{r},t)$, and generalized local-time wavevector, $k_\alpha(\mathbf{r},\tau)$, as equations (C15) and (C16), and substitute equations (C15) and (C16) into (D10), the final closed-form amplitude-phase expression is given by:

$$E = i \frac{\alpha(\alpha+1) w_0^{\alpha+1} r A_\alpha(\mathbf{r},\tau)}{2^\alpha z_0^{\alpha+1} w^{\alpha+1} \left(1+\frac{r^2}{2w^2}\right)^{\alpha+2}} \exp\left\{i[k_\alpha(\mathbf{r},\tau) + (\alpha+1)\phi(z)]\right\} \quad \text{(D11)}$$

Note that when $\alpha = 1$, the amplitude-phase expression of high-order flying doughnut pulse of equation (D11) will be reduced into the amplitude-phase expression of fundamental flying doughnut pulse as equation (B9). Also, when the index of α goes higher, the energy of the pulse would be smaller because $\frac{\alpha(\alpha+1)}{2^\alpha} \to 0$ when α goes to infinity, that is consistent with the meaning in the finite-energy assumption in the conventional EDEPT method.

Supplementary Information E: Phase velocity of high-order few-cycle pulses

Before deriving the group velocity and phase velocity, we firstly simplify the amplitude-phase expressions of high-order FP and FD pulses, for neglecting the constant factors in amplitude and considering the propagation within the Rayleigh range:

$$E^{(\mathrm{FP})}=\frac{1}{w^{\alpha}\left(1+\dfrac{r^2}{2w^2}\right)^{\alpha+2}}\frac{\left(q_1^2+\tau^2\right)^{(\alpha-1)/2}}{(T^2+1)^{(\alpha+2)/2}}\exp\left\{i\left[(\alpha-1)\tan^{-1}\left(\frac{\tau}{q_1}\right)+(\alpha+2)\tan^{-1}T+\alpha\tan^{-1}\left(\frac{z}{z_0}\right)\right]\right\} \quad \text{(E1)}$$

$$E^{(\mathrm{FD})}=\frac{r}{w^{\alpha+1}\left(1+\dfrac{r^2}{2w^2}\right)^{\alpha+2}}\frac{\left(q_1^2+\tau^2\right)^{(\alpha-1)/2}}{(T^2+1)^{(\alpha+2)/2}}\exp\left\{i\left[(\alpha-1)\tan^{-1}\left(\frac{\tau}{q_1}\right)+(\alpha+2)\tan^{-1}T+(\alpha+1)\tan^{-1}\left(\frac{z}{z_0}\right)\right]\right\} \quad \text{(E2)}$$

For evaluating the phase velocity, that the speed of the traveling of constant-phase surface, we focused on the constant phase equations for FD and FP pulses:

$$\varphi^{(\mathrm{FP})}=(\alpha-1)\tan^{-1}\left(\frac{\tau}{q_1}\right)+(\alpha+2)\tan^{-1}T+\alpha\tan^{-1}\left(\frac{z}{z_0}\right)=\text{const.} \quad \text{(E3)}$$

$$\varphi^{(\mathrm{FD})}=(\alpha-1)\tan^{-1}\left(\frac{\tau}{q_1}\right)+(\alpha+2)\tan^{-1}T+\left(\alpha+1\right)\tan^{-1}\left(\frac{z}{z_0}\right)=\text{const.} \quad \text{(E4)}$$

To solve the phase velocity of FP pulse, we make the differential for both z and t terms of the equation (E3):

$$(\alpha-1)\frac{1}{1+\left(\dfrac{\tau}{q_1}\right)^2}\frac{1}{q_1}\delta\tau+(\alpha+2)\frac{1}{1+T^2}\delta T+\alpha\frac{1}{1+\left(\dfrac{z}{z_0}\right)^2}\frac{1}{z_0}\delta z=0 \quad \text{(E5)}$$

For the effective pulse region, $r \ll R$, thus the $r^2/(2R)$ is extremely small and can be ignored. For the near-axis region of $r<\sqrt{2}w$, the term $r^2/(2w^2)$ is slowly-variant function versus z, and based on equation (A10), the differential of T can be simplified as:

$$T=\frac{-\left(\tau+\dfrac{r^2}{2R}\right)}{q_1\left(1-\dfrac{r^2}{2w^2}\right)}\approx\frac{-\tau}{q_1}\left(1-\frac{r^2}{2w^2}\right)\Rightarrow\delta T=-\frac{1}{q_1}\left(1-\frac{r^2}{2w^2}\right)\delta\tau \quad \text{(E6)}$$

Substituting (E6) into (E5) and based on the short pulse assumption, thus the factors of the square of local time can be regarded as zero, $(\tau/q_1)^2=T^2\approx 0$, then equation (E5) is derived as:

$$(\alpha-1)\frac{1}{q_1}\delta\tau-(\alpha+2)\frac{1}{q_1}\left(1-\frac{r^2}{2w^2}\right)\delta\tau+\alpha\frac{1}{1+\left(\dfrac{z}{z_0}\right)^2}\frac{1}{z_0}\delta z=0 \quad \text{(E7)}$$

After simplification:

$$\left[\frac{(\alpha+2)r^2}{2q_1w^2}-\frac{3}{q_1}\right]\delta\tau+\alpha\frac{1}{1+\left(\frac{z}{z_0}\right)^2}\frac{1}{z_0}\delta z=0 \tag{E8}$$

Substitute the $\tau=z-ct$, i.e. $\delta\tau=\delta z-c\delta t$ into (E6):

$$\left[\frac{(\alpha+2)r^2}{2q_1w^2}-\frac{3}{q_1}\right]c\delta t+\left[\frac{\alpha}{z_0}\frac{1}{1+\left(z/z_0\right)^2}+\frac{(\alpha+2)r^2}{2q_1w^2}-\frac{3}{q_1}\right]\delta z=0 \tag{E9}$$

Then the phase velocity of FP pulse can be expressed as:

$$\begin{aligned} v_{\mathrm{p}}^{(\mathrm{FP})}\left(r,z\right)&=\frac{\delta z}{\delta t}=-\frac{\left[\frac{(\alpha+2)r^2}{2q_1w^2}-\frac{3}{q_1}\right]c}{\frac{\alpha}{z_0}\frac{1}{1+\left(z/z_0\right)^2}+\frac{(\alpha+2)r^2}{2q_1w^2}-\frac{3}{q_1}} \\ &=\frac{c}{1-\frac{\alpha}{z_0}\frac{1}{1+\left(z/z_0\right)^2}\Big/\left[\frac{3}{q_1}-\frac{(\alpha+2)r^2}{2q_1w^2}\right]} \end{aligned} \tag{E10}$$

Similarly, the phase velocity of FD pulse can be derived as:

$$v_{\mathrm{p}}^{(\mathrm{FD})}\left(r,z\right)=\frac{c}{1-\frac{\alpha+1}{z_0}\frac{1}{1+\left(z/z_0\right)^2}\Big/\left[\frac{3}{q_1}-\frac{(\alpha+2)r^2}{2q_1w^2}\right]} \tag{E11}$$

The on-axis phase velocities of FP and FD pulses are given by:

$$v_{\mathrm{p}}^{(\mathrm{FP})}\left(z\right)=\frac{c}{1-\frac{\alpha q_1}{3z_0}\frac{1}{1+\left(z/z_0\right)^2}} \tag{E12}$$

Similarity, the on-axis phase velocity of FD pulse can be expressed as:

$$v_{\mathrm{p}}^{(\mathrm{FD})}\left(z\right)=\frac{c}{1-\frac{(\alpha+1)q_1}{3z_0}\frac{1}{1+\left(z/z_0\right)^2}} \tag{E13}$$

Supplementary Information F: Group velocity of high-order few-cycle pulses

For evaluating the group velocity, that the speed of the traveling of amplitude envelope, we focused on the amplitude expression. After substituting T and τ, the amplitude expressions for FD and FP pulses are given as:

$$A^{(\mathrm{FP})} = \frac{\left[q_1^2 + (z-ct)^2\right]^{(\alpha-1)/2}}{w^{\alpha}\left[\left(z-ct+\dfrac{r^2}{2R}\right)^2 + q_1^2\left(1+\dfrac{r^2}{2w^2}\right)^2\right]^{(\alpha+2)/2}} \tag{F1}$$

$$A^{(\mathrm{FD})} = \frac{r\left[q_1^2 + (z-ct)^2\right]^{(\alpha-1)/2}}{w^{\alpha+1}\left[\left(z-ct+\dfrac{r^2}{2R}\right)^2 + q_1^2\left(1+\dfrac{r^2}{2w^2}\right)^2\right]^{(\alpha+2)/2}} \tag{F2}$$

For characterizing the velocity, we should firstly solve the trajectory of the maximum of the amplitude envelope, and then solve the traveling velocity of the trajectory. For both equations (F1) and (F2), if we ignore the numerator term (set α=1 in the numerator term), it is easy to find the trajectory yields the maximum of the amplitude, i.e. the trajectory yields the minimum of the denominator:

$$F(r,z,t) = z-ct+\frac{r^2}{2R} = z-ct+\frac{zr^2}{2\left(z^2+z_0^2\right)} = 0 \tag{F3}$$

Then we can derive the group velocity for the case of α=1, that is available for both FP and FD cases. Making deferential for the z and t in equation (F3), we get:

$$\delta z = c\delta t + \frac{z^2-z_0^2}{2\left(z^2+z_0^2\right)}r^2\delta z \tag{F4}$$

Thus the group velocity for the fundamental FP and FD pulses can be given by

$$v_g^{(\alpha=1)} = \frac{\delta z}{\delta t} = \frac{c}{1-\dfrac{z^2-z_0^2}{2\left(z^2+z_0^2\right)^2}r^2} \tag{F5}$$

Hereinafter, we consider the general case of α>1, the key issue is found the maximum value point of the function of $f(\tau) = \left(q_1^2+\tau^2\right)^{(\alpha-1)/2} \Big/ \left[\left(\tau+d_1\right)^2 + d_2^2\right]^{(\alpha+2)/2}$, where the $d_1 = \dfrac{r^2}{2R}$ and $d_2 = q_1\left(1+\dfrac{r^2}{2w^2}\right)$ are regarded as z-independent because they are very slowly-variant. Both FP and FD pulses with the same order of α have the same maximum value point as the function of $f(\tau)$, in order to solve it, we calculate the differential of $f(\tau)$:

$$\begin{aligned}\frac{\mathrm{d}}{\mathrm{d}\tau}f(\tau) &= -\frac{(\alpha+2)\left(\tau+d_1\right)\left(q_1^2+\tau^2\right)^{(\alpha-1)/2}}{\left[\left(\tau+d_1\right)^2+d_2^2\right]^{(\alpha+2)/2+1}} + \frac{(\alpha-1)\tau\left(q_1^2+\tau^2\right)^{(\alpha-1)/2-1}}{\left[\left(\tau+d_1\right)^2+d_2^2\right]^{(\alpha+2)/2}} \\ &= \frac{(\alpha-1)\tau\left[\left(\tau+d_1\right)^2+d_2^2\right] - (\alpha+2)\left(\tau+d_1\right)\left(q_1^2+\tau^2\right)}{\left[\left(\tau+d_1\right)^2+d_2^2\right]^{(\alpha+2)/2+1}}\left(q_1^2+\tau^2\right)^{(\alpha-1)/2-1}\end{aligned} \tag{F6}$$

The maximum value position fulfills the zero point of $\frac{d}{d\tau} f(\tau) = 0$, equivalently

$$(\alpha - 1)\tau\left[\left(\tau + d_1\right)^2 + d_2^2\right] = (\alpha + 2)\left(\tau + d_1\right)\left(q_1^2 + \tau^2\right) \tag{F7}$$

It is obvious that the zero point is $\tau = -d_1$ for $\alpha = 1$. Thus, we can assume the zero point as $\tau = -d_1 - d_\alpha$ where we assume $d_\alpha < d_1$ is an perturbation quantity, for general case of $\alpha \geq 1$, then the d_α fulfills:

$$(\alpha - 1)\left(d_1 + d_\alpha\right)\left[d_\alpha^2 + d_2^2\right] = (\alpha + 2)d_\alpha\left[q_1^2 + \left(d_1 + d_\alpha\right)^2\right] \tag{F8}$$

Under the limit of $r \ll \sqrt{2}w$, and d_α is smaller than q_1 and q_2, we ignore the higher-order small value of d_α and get

$$(\alpha - 1)\left(d_1 + d_\alpha\right)d_2^2 = (\alpha + 2)d_\alpha\left(q_1^2 + d_1^2\right) \tag{F9}$$

And solve the d_α as

$$\begin{aligned} d_\alpha &= \frac{(\alpha - 1)d_1 d_2^2}{(\alpha + 2)\left(q_1^2 + d_1^2\right) - (\alpha - 1)d_2^2} \\ &= \frac{(\alpha - 1)q_1^2 \dfrac{r^2}{2R}\left(1 + \dfrac{r^2}{2w^2}\right)^2}{(\alpha + 2)\left[q_1^2 + \left(\dfrac{r^2}{2R}\right)^2\right] - (\alpha - 1)q_1^2\left(1 + \dfrac{r^2}{2w^2}\right)^2} \end{aligned} \tag{F10}$$

Based on our assumption, this equation is only available under the limit of $r \ll w$. For extending the effective evaluation, we calculate the various-order differential to r^2 on the axis:

$$d_\alpha{}' = \left.\frac{\mathrm{d}d_\alpha}{\mathrm{d}(r^2)}\right|_{r^2=0} = \frac{(\alpha - 1)}{6R}; \quad d_\alpha{}'' = \left.\frac{\mathrm{d}^2 d_\alpha}{\mathrm{d}(r^2)^2}\right|_{r^2=0} = \frac{(\alpha - 1)(\alpha + 2)}{9Rw^2}; \quad \cdots\cdots \tag{F11}$$

Therefore, the maximum value trajectory of the amplitude is expressed as

$$\begin{aligned} F_\alpha(r,z,t) &= z - ct + \frac{r^2}{2R} + d_\alpha = z - ct + \frac{r^2}{2R} + \left(d_\alpha{}' r^2 + \frac{d_\alpha{}''}{2} r^4 + \cdots\cdots\right) \\ &\approx ct + \frac{r^2}{2R} + \frac{(\alpha - 1)}{6R} r^2 = ct + \frac{\alpha + 2}{6R} r^2 = 0 \end{aligned} \tag{F12}$$

Making deferential for the z and t in equation (F12), we get:

$$\delta z = c\delta t + \frac{\alpha + 2}{6}\frac{z^2 - z_0^2}{z^2 + z_0^2} r^2 \delta z \tag{F13}$$

Thus the group velocity for the high-order FP and FD pulses can be given by

$$v_g^{(\alpha)} = \frac{\delta z}{\delta t} = \frac{c}{1 - \dfrac{\alpha + 2}{6}\dfrac{z^2 - z_0^2}{z^2 + z_0^2} r^2} \tag{F14}$$

Numerical verification of Maxwell's equations for high-order few-cycle pulses.

To verify the reliability of our approach, we compared the results of the original equations derived from EDEPT method, the local-time amplitude-phase formation, and the numerical results by directly solving Maxwell's equations. The results from these various calculations indeed show the same results:

	Original equations	Amplitude-phase formation	Numerical solution of COMSOL
$\alpha = 1$	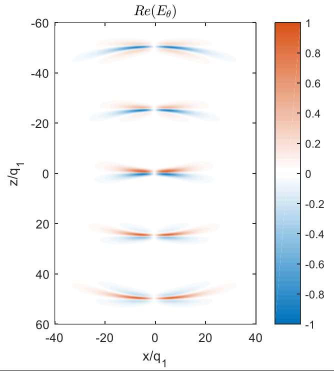	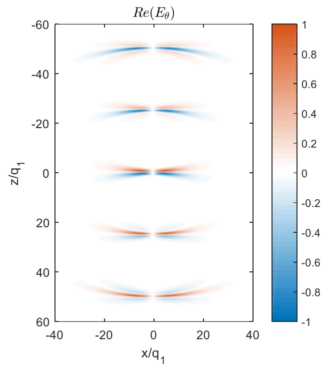	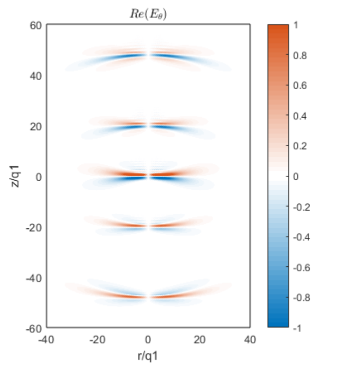
$\alpha = 3$	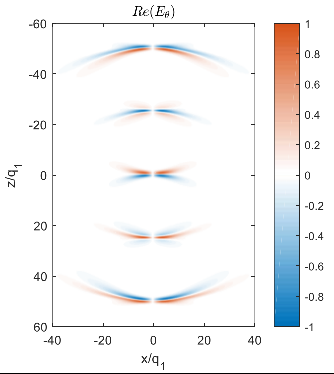	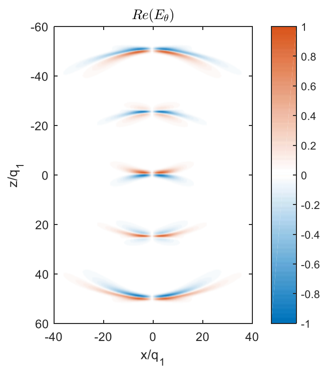	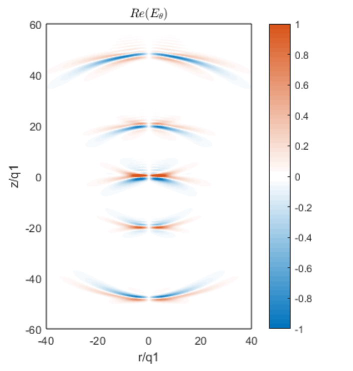
$\alpha = 6$	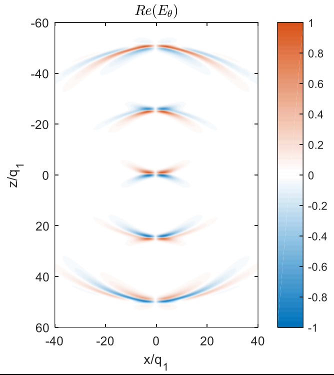	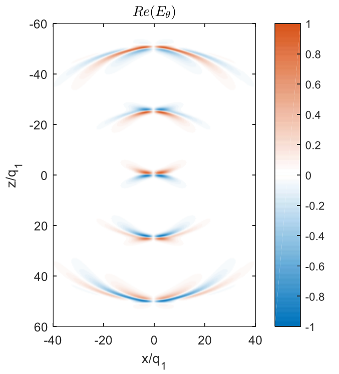	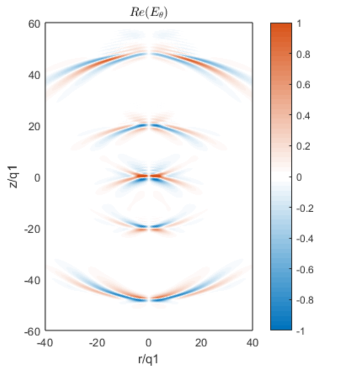
$\alpha = 10$	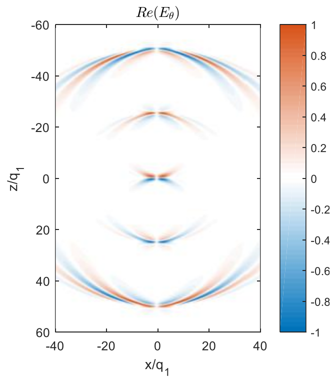	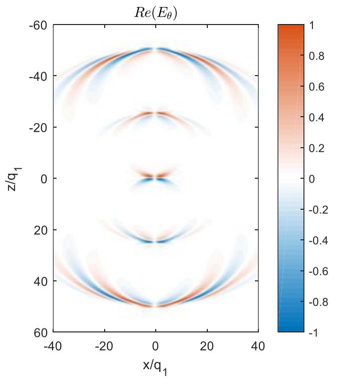	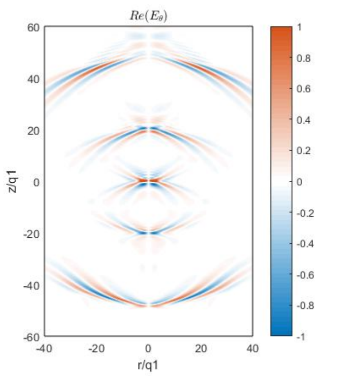

Supplementary Information G:
The trajectories of equation (F3), $F(r,z,t) = 0$, at various times are shown as blue lines in Figs. S1a and S1b, together with the envelope distributions of FP and FD pulses correspondingly. The trajectories of equation (F12), $F_\alpha(r,z,t) = 0$, for the 4th-order (α=4) at FP and FD pulses at various times are plot-ted as blue lines in Figs. S1c and S1d, which can correctly reveal the location of pulse envelope.

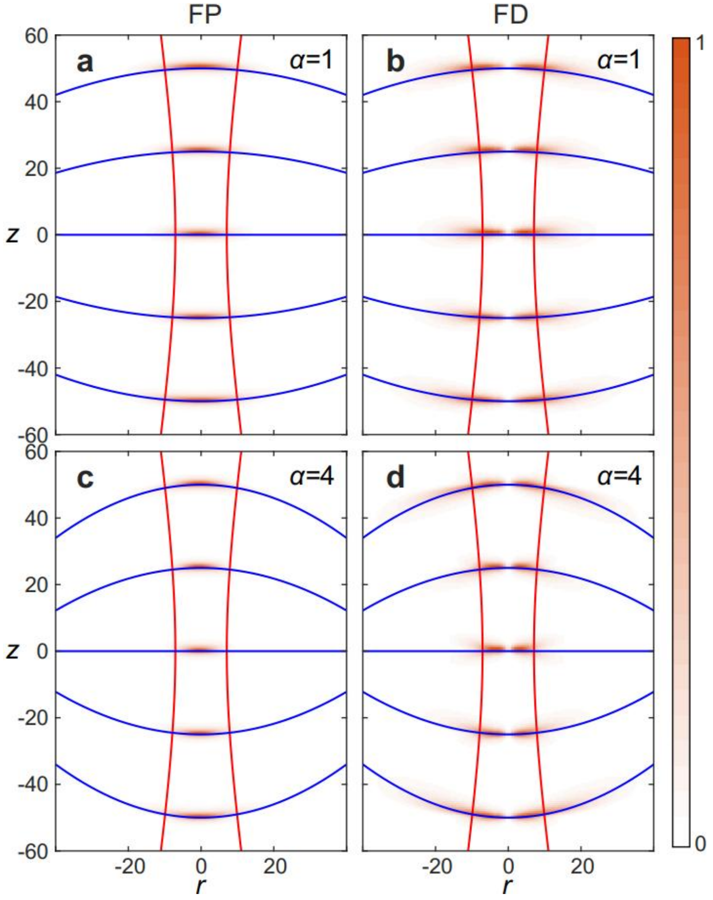

Fig. S1 Amplitude envelope of high-order few-cycle pulses: a-d, The amplitude envelope distributions of fundamental FP (a) and FD (b) pulses and higher-order (α= 4) FP (c) and FD (d) at various times $t = 0$, $\pm z_0/(2c)$, and $\pm z_0/c$. The red lines mark the trajectories of $w(z)$ and the blue lines mark the centroid surfaces of $F_\alpha(r,z,t) = 0$.

Supplementary Information H: EDEPT Synthesis of p-stretched FP/FD Pulses

1. Coordinates, slow variables, and geometry

We use cylindrical coordinates (ρ, θ, z) and time t. Define

$$\tau = z - ct, \qquad \sigma = z + ct.$$

For near-axis comparisons we collect the standard Gaussian-beam geometry:

$$\frac{1}{R(z)} = \frac{z}{z^2 + z_0^2}, \qquad w(z) = w_0\sqrt{1 + (z/z_0)^2}, \qquad \phi'(z) = \frac{1}{z_0\,[1 + (z/z_0)^2]},$$

with $z_0 = \frac{q_2}{2}$, $w_0 = \sqrt{\frac{q_1 q_2}{2}}$. These are used later for Rayleigh-limit reductions only.

Introduce

$$A := q_1 + \mathrm{i}\tau, \qquad \frac{1}{A} = \frac{q_1}{q_1^2 + \tau^2} - \mathrm{i}\,\frac{\tau}{q_1^2 + \tau^2}$$

(valid for all (z, t)).

2. EDEPT kernel, weight, and generator

Kernel

$$\Phi_a(\boldsymbol{r}, t) = \frac{1}{A}\,\exp\!\left[-a\,\frac{\rho^2}{A}\right]\,\exp(\mathrm{i}a\,\sigma), \qquad a \geq 0. \tag{H1}$$

Shifted-Gamma weight and closed generator

Let

$$W_\alpha(a) = \frac{(a - \frac{1}{q_3})^{\alpha-1}}{\Gamma(\alpha)}\,\exp\Big[-q_2\Big(a - \tfrac{1}{q_3}\Big)\Big]\,H\Big(a - \tfrac{1}{q_3}\Big). \tag{H2}$$

Define $s := \rho^2/A - \mathrm{i}\sigma$ and $B := \rho^2 + A(q_2 - \mathrm{i}\sigma) = A(q_2 + s)$. Then the generator is

$$f_\alpha(\boldsymbol{r}, t) = f_0 \int_0^\infty \Phi_a\,W_\alpha\,da = \frac{f_0}{A}\,\frac{\exp(-s/q_3)}{(q_2 + s)^\alpha} = f_0\,A^{\alpha-1}\,\mathrm{e}^{-s/q_3}\,B^{-\alpha}. \tag{H3}$$

3. Hertz potentials and FP/FD fields

We use the magnetic-type Hertz potential:

$$\boldsymbol{E} = -\mu_0\,\partial_t(\nabla\times\boldsymbol{\Pi}), \qquad \boldsymbol{H} = \nabla\times(\nabla\times\boldsymbol{\Pi}).$$

FP: $\boldsymbol{\Pi} = \hat{\boldsymbol{x}} f_\alpha$ (full vector form)

Since $f_\alpha = f_\alpha(\rho, z, t)$ and $\rho = \sqrt{x^2+y^2}$,

$$\nabla\times(\hat{\boldsymbol{x}} f_\alpha) = (0, \partial_z f_\alpha, -\partial_y f_\alpha), \quad \partial_y f_\alpha = \frac{y}{\rho}\,\partial_\rho f_\alpha.$$

Thus

$$E_y^{(\mathrm{FP})} = -\mu_0\,\partial_t\partial_z f_\alpha, \qquad E_z^{(\mathrm{FP})} = +\mu_0\,\partial_t\partial_y f_\alpha = \frac{y}{\rho}\,\mu_0\,\partial_t\partial_\rho f_\alpha. \tag{H4}$$

FD: $\boldsymbol{\Pi} = \hat{\boldsymbol{z}} f_\alpha$

For axisymmetric f_α, $\nabla\times(\hat{\boldsymbol{z}} f_\alpha) = (0, -\partial_\rho f_\alpha, 0) = -\hat{\boldsymbol{\theta}}\,\partial_\rho f_\alpha$, so

$$E_\theta^{(\mathrm{FD})} = +\mu_0\,\partial_t\partial_\rho f_\alpha.$$

Closed forms (exact)

With $B = \rho^2 + A(q_2 - \mathrm{i}\sigma)$,

$$\boxed{E_y^{(\mathrm{FP})}(\boldsymbol{r},t) = -\alpha(\alpha+1)\,f_0\sqrt{\frac{\mu_0}{\varepsilon_0}}\,A^{\alpha-1}\,\frac{A^2 - (q_2 - \mathrm{i}\sigma)^2}{B^{\alpha+2}}} \tag{H5}$$

$$\boxed{E_\theta^{(\mathrm{FD})}(\boldsymbol{r},t) = +\alpha(\alpha+1)\,\mathrm{i}\,f_0\sqrt{\frac{\mu_0}{\varepsilon_0}}\,A^{\alpha-1}\,\frac{\rho(A + q_2 - \mathrm{i}\sigma)}{B^{\alpha+2}}} \tag{H6}$$

Note: $A + q_2 - \mathrm{i}\sigma = q_1 + q_2 - 2\mathrm{i}ct$ (independent of z). From (H4) and the FD result,

$$E_z^{(\mathrm{FP})} = \frac{y}{\rho}\,E_\theta^{(\mathrm{FD})}.$$

4. Exact p-stretch (spectral phase mask)

Define $S_p(a) = \sum_m w_m\,\mathrm{e}^{-\mathrm{i}a\Delta_m}$ with $\sum_m w_m = 1$ and $W_{\alpha,p} = S_p W_\alpha$. Then

$$f_{\alpha,p}(\boldsymbol{r},t) = \sum_m w_m\,f_\alpha\big(\rho, z;\, \sigma - \Delta_m\big),$$

and

$$E_y^{(\mathrm{FP},p)} = \sum_m w_m\Big[-\alpha(\alpha+1)f_0\sqrt{\tfrac{\mu_0}{\varepsilon_0}}\,A^{\alpha-1}\frac{A^2-\big(q_2-\mathrm{i}(\sigma-\Delta_m)\big)^2}{\big(\rho^2+A(q_2-\mathrm{i}(\sigma-\Delta_m))\big)^{\alpha+2}}\Big], \tag{H7}$$

$$E_\theta^{(\mathrm{FD},p)} = \sum_m w_m\Big[+\alpha(\alpha+1)\mathrm{i}f_0\sqrt{\tfrac{\mu_0}{\varepsilon_0}}\,A^{\alpha-1}\frac{\rho\big(A+q_2-\mathrm{i}(\sigma-\Delta_m)\big)}{\big(\rho^2+A(q_2-\mathrm{i}(\sigma-\Delta_m))\big)^{\alpha+2}}\Big]. \tag{H8}$$

Both numerator and denominator shift with $\sigma \longrightarrow \sigma-\Delta_m$. This stretch does not modify the per-term phase law.

5. Amplitude–phase factorization

Any complex component can be written as $E = \mathcal{A}\,\exp(\mathrm{i}\Phi)$ with $\mathcal{A} = |E|$ and $\Phi = \operatorname{Arg}E$. Introduce

$$S := q_2 - \mathrm{i}\sigma, \qquad N_\pm := A \pm S, \qquad B = \rho^2 + AS.$$

Then

$$E_y^{(\mathrm{FP})} = \mathcal{A}^{(\mathrm{FP})}\,\mathrm{e}^{\mathrm{i}\Phi^{(\mathrm{FP})}} \tag{H9}$$

$$\mathcal{A}^{(\mathrm{FP})} = |-C_{FP}|\,|A|^{\alpha-1}\frac{|N_-|\,|N_+|}{|B|^{\alpha+2}}, \tag{H10}$$

$$\Phi^{(\mathrm{FP})} = \operatorname{Arg}(-C_{FP}) + (\alpha-1)\operatorname{Arg}A + \operatorname{Arg}N_- + \operatorname{Arg}N_+ - (\alpha+2)\operatorname{Arg}B, \tag{H11}$$

$$E_\theta^{(\mathrm{FD})} = \mathcal{A}^{(\mathrm{FD})}\,\mathrm{e}^{\mathrm{i}\Phi^{(\mathrm{FD})}} \tag{H12}$$

$$\mathcal{A}^{(\mathrm{FD})} = |C_{FD}|\,|A|^{\alpha-1}\frac{\rho\,|N_+|}{|B|^{\alpha+2}}, \tag{H13}$$

$$\Phi^{(\mathrm{FD})} = \operatorname{Arg}(\mathrm{i}C_{FD}) + (\alpha-1)\operatorname{Arg}A + \operatorname{Arg}N_+ - (\alpha+2)\operatorname{Arg}B, \tag{H14}$$

with $C_{FP} = C_{FD} = \alpha(\alpha+1)f_0\sqrt{\mu_0/\varepsilon_0}$. On axis ($\rho = 0$), $B = A\,S$ so

$$\Phi^{(\mathrm{FP})}_{\rho=0} = \operatorname{Arg}(-C_{FP}) - 3\operatorname{Arg}A + \operatorname{Arg}N_- + \operatorname{Arg}N_+ - (\alpha+2)\operatorname{Arg}S,\ \Phi^{(\mathrm{FD})}_{\rho=0} = \operatorname{Arg}(\mathrm{i}C_{FD}) - 3\operatorname{Arg}A + \operatorname{Arg}N_+ - (\alpha+2)\operatorname{Arg}S. \tag{H15}$$

6. FD–FP Gouy/Rayleigh coefficient: detailed derivation

We now extract the coefficient multiplying the Rayleigh/Gouy phase $\phi(z) = \arctan(z/z_0)$ in the near-axis regime. The key point is to evaluate z-variations along a local-time slice $T = \text{const}$ (in particular, the pulse peak $T = 0$), which is the natural choice for ultrashort pulsed beams.

Step 1: T-slice kinematics on axis

Recall that

$$T(\rho,z,t) = \frac{ct-\chi(\rho,z)}{\Gamma\,\eta(\rho,z)}, \qquad \chi = z + \frac{\rho^2}{2R(z)}, \qquad \eta = 1 + \frac{\rho^2}{p\,w^2(z)}, \qquad \Gamma = \frac{(\alpha+2)q_1}{2}.$$

On axis ($\rho = 0 \Rightarrow \eta = 1$), the constraint $dT = 0$ gives

$$\frac{d(ct)}{dz} = \frac{d\chi}{dz} = 1 \quad\Rightarrow\quad \frac{dt}{dz} = \frac{1}{c}.$$

Therefore, along a fixed T slice,

$$\frac{d\tau}{dz} = \frac{d(z-ct)}{dz} = 1 - c\frac{dt}{dz} = 0, \qquad \frac{d\sigma}{dz} = \frac{d(z+ct)}{dz} = 1 + c\frac{dt}{dz} = 2.$$

Hence $A = q_1 + \mathrm{i}\tau$ is *constant* on the slice, while $S = q_2 - \mathrm{i}\sigma$ varies with z through $\sigma(z)$.

Step 2: z-derivatives of the on-axis phase building blocks

Using (H15) and the results above:

$$\frac{d}{dz}\operatorname{Arg} A = 0, \qquad \frac{d}{dz}\operatorname{Arg} S = \frac{d}{dz}\big[-\arctan(\sigma/q_2)\big] = -\frac{1}{q_2}\frac{d\sigma/dz}{1+(\sigma/q_2)^2} = -\frac{2/q_2}{1+4z^2/q_2^2} = -\phi'(z).$$

For the remaining factors,

$$N_+ = A + S = (q_1+q_2) - \mathrm{i}\,2ct, \qquad N_- := A - S = (q_1 - q_2) + \mathrm{i}\,2z,$$

so that along a T-slice

$$\frac{d}{dz}\operatorname{Arg} N_+ = \frac{\partial \operatorname{Arg} N_+}{\partial(ct)}\frac{d(ct)}{dz} = -\frac{2}{q_1+q_2}\frac{1}{1+4(ct)^2/(q_1+q_2)^2},$$

and at the peak $T = 0$ (hence $ct \simeq z$ near axis),

$$\frac{d}{dz}\operatorname{Arg} N_+ = -\frac{2}{q_1+q_2}\frac{1}{1+4z^2/(q_1+q_2)^2}.$$

Similarly,

$$\frac{d}{dz}\operatorname{Arg} N_- = \frac{2}{q_1-q_2}\frac{1}{1+4z^2/(q_1-q_2)^2}.$$

Step 3: Effective Gouy coefficients

Define the Gouy coefficient functions by normalizing the z-phase gradient to the Gaussian $\phi'(z) = \frac{2/q_2}{1+4z^2/q_2^2}$:

$$A_3^{(\mathrm{FD})}(z) := \frac{d\Phi^{(\mathrm{FD})}_{\rho=0}/dz}{\phi'(z)} = (\alpha+2) - \kappa_+(z), \tag{H16}$$

$$A_3^{(\mathrm{FP})}(z) := \frac{d\Phi^{(\mathrm{FP})}_{\rho=0}/dz}{\phi'(z)} = (\alpha+2) - \kappa_+(z) - \kappa_-(z), \tag{H17}$$

where the positive correction factors

$$\kappa_+(z) = \frac{q_2}{q_1+q_2}\frac{1+4z^2/q_2^2}{1+4z^2/(q_1+q_2)^2}, \qquad \kappa_-(z) = \frac{q_2}{q_2-q_1}\frac{1+4z^2/q_2^2}{1+4z^2/(q_1-q_2)^2}. \tag{H18}$$

At focus $z = 0$:

$$A_3^{(\mathrm{FD})}(0) = \alpha + 2 - \frac{q_2}{q_1+q_2} = \alpha + 1 + \frac{q_1}{q_1+q_2}, \qquad A_3^{(\mathrm{FP})}(0) = \alpha + 2 - \frac{q_2}{q_1+q_2} - \frac{q_2}{q_2-q_1}.$$

Thus the difference is

$$A_3^{(\mathrm{FD})}(0) - A_3^{(\mathrm{FP})}(0) = \kappa_-(0) = \frac{q_2}{q_2 - q_1} = 1 + O\left(\frac{q_1}{q_2}\right). \tag{H19}$$

Leading-order result (Rayleigh/near-axis, $q_2 \gg q_1$). For the usual experimental scaling $q_2 \gg q_1$ and $|z| \lesssim z_0$ we have

$$\kappa_\pm(z) = 1 + O\left(\frac{q_1}{q_2}, \frac{z^2}{z_0^2}\right).$$

Hence

$$A_3^{(\mathrm{FD})} \approx \alpha + 1, \qquad A_3^{(\mathrm{FP})} \approx \alpha,$$

i.e. the FD Gouy coefficient exceeds the FP one by one unit to leading order.

p-stretch invariance. The p-stretch acts as $\sigma \to \sigma - \Delta_m$ termwise and does not modify the derivatives above with respect to z (at fixed T), hence it does not change $A_3^{(\mathrm{FD})}$ or $A_3^{(\mathrm{FP})}$.

7. Phase and group velocities

We parameterize the local phase as

$$\Phi(\rho, z, t) = A_1 \arctan\frac{\tau_p}{q_1} + A_2 \arctan T + A_3 \arctan\frac{z}{z_0}, \qquad (A_1, A_2, A_3) = \begin{cases} (\alpha - 1,\ \alpha + 2,\ \alpha), & \text{FP}, \\ (\alpha - 1,\ \alpha + 2,\ \alpha + 1), & \text{FD}, \end{cases} \tag{H20}$$

with $\tau_p := \tau/p$ and

$$T(\rho, z, t) = \frac{ct - \chi(\rho, z)}{\Gamma\,\eta(\rho, z)}, \quad \chi = z + \frac{\rho^2}{2R(z)}, \quad \eta = 1 + \frac{\rho^2}{p\,w^2(z)}, \quad \Gamma = \frac{(\alpha + 2)q_1}{2}.$$

Derivatives:

$$\partial_t \tau_p = -c/p, \quad \partial_z \tau_p = +1/p, \quad \partial_t T = \frac{c}{\Gamma\eta}, \quad \partial_z T = -\frac{\chi'}{\Gamma\eta} - T\,\frac{\eta'}{\eta},$$

$$\chi' = 1 - \frac{\rho^2}{2}\frac{R'}{R^2}, \qquad \eta' = -\frac{2\rho^2}{p}\frac{w'}{w^3}.$$

Exact local-gradient form for v_p

From $v_p = -\partial_t\Phi/\partial_z\Phi$,

$$\boxed{v_p(\rho, z, t) = \frac{c\,U - \dfrac{A_2}{1 + T^2}\,\partial_t T}{U + \dfrac{A_2}{1 + T^2}\,\partial_z T + A_3\,\phi'(z)}, \qquad U := \frac{A_1}{p\,q_1}\frac{1}{1 + (\tau_p/q_1)^2}.} \tag{H21}$$

Near-axis (Rayleigh) reduction

In the Rayleigh range and $r \ll w$,

$$T \simeq -\frac{2p}{\alpha+2}\frac{\tau_p}{q_1}\left(1-\frac{\rho^2}{2w^2}\right), \tag{H22}$$

leading to

$$v_p^{(\mathrm{FP})}(r,z;\alpha) \simeq \frac{\left[\frac{\alpha+2}{2q_1}\frac{r^2}{w^2}-\frac{3}{q_1}\right]c}{\frac{\alpha}{z_0[1+(z/z_0)^2]}+\frac{\alpha+2}{2q_1}\frac{r^2}{w^2}-\frac{3}{q_1}}, \tag{H23}$$

$$v_p^{(\mathrm{FD})}(r,z;\alpha) \simeq \frac{\left[\frac{\alpha+2}{2q_1}\frac{r^2}{w^2}-\frac{3}{q_1}\right]c}{\frac{\alpha+1}{z_0[1+(z/z_0)^2]}+\frac{\alpha+2}{2q_1}\frac{r^2}{w^2}-\frac{3}{q_1}}. \tag{H24}$$

Group velocity from the T-level set

From $dT = 0$,

$$v_g(\rho,z;T) = \frac{dz}{dt} = -\frac{\partial_t T}{\partial_z T} = \frac{c}{1-\frac{\rho^2}{2}\frac{R'}{R^2}-\Gamma\frac{2\rho^2}{p}\frac{w'(z)}{w^3(z)}T}, \qquad \Gamma = \frac{(\alpha+2)q_1}{2}. \tag{H25}$$

At the envelope peak $T = 0$,

$$v_g^{\mathrm{peak}}(\rho,z) = \frac{c}{1-\frac{\rho^2}{2}\frac{R'}{R^2}}, \tag{H26}$$

independent of α and p. This peak velocity should be compared with the centroid-based expression $v_g^{(\alpha)}(r,z)$ given in Eq. (22) of the main text and derived in Supplementary Information Sec. F by expanding the amplitude envelopes $A_\alpha^{(\mathrm{FP})}$ and $A_\alpha^{(\mathrm{FD})}$ and solving for the motion of the centroid surface $F_\alpha(r,t) = 0$. For the fundamental order $\alpha = 1$ the centroid and the $T = 0$ level set coincide, so the two definitions of group velocity are identical. For higher orders $\alpha > 1$ the envelopes develop a weak temporal asymmetry, and the centroid surface is slightly displaced relative to the $T = 0$ surface. In that case Eq. (22) can be viewed as providing small α–dependent corrections to the universal peak law Eq. (23), arising purely from the choice of tracking the centroid rather than the envelope maximum. In all parameter ranges used in Fig. 5 the two prescriptions agree to within a few percent, so the identification of subluminal and superluminal regions is not affected by this distinction.

Forward propagation. All formulas above were written with the kernel phase $e^{ia\sigma}$. If forward propagation along $+z$ is desired, replace $\sigma \mapsto -\tau$ everywhere (e.g. $S \to q_2 + i\tau$, $B \to \rho^2 + A(q_2 + i\tau)$); the conclusions in gouy-derivation remain unchanged.

Spectral characterization and the role of p

Figures S1 (Flying Donut; FD) and S2 (Flying Pancake; FP) display one-sided, baseband, de-chirped envelope power spectra $S(\Omega) \propto \left|\mathcal{F}_t\{E_{\mathrm{dc}}(t)\}\right|^2$ evaluated at the transverse position of maximum electric-field magnitude (the ring maximum at $z = 0$). Here $E_{\mathrm{dc}}(t)$ denotes the complex field after removing the fast optical phase so that only the temporal envelope remains; the ordinate is normalized to unity within

the plotted frequency window, and the abscissa is the angular frequency Ω (rad/s). Showing only $\Omega \geq 0$ (one-sided) avoids duplicating the Hermitian mirror of real signals.

Increasing p slows the envelope in time (larger $t_{1/2}$) and therefore narrows the baseband spectrum; decreasing p speeds it up and broadens the spectrum. In the large-p limit the de-chirped envelope approaches a quasi-monochromatic (CW-like) behavior, while small p corresponds to a broadband, ultrashort-pulse-like regime.

The spectra were computed from long time records tapered with a smooth window to suppress spectral leakage; zero-padding refines the frequency sampling but does not alter bandwidths. The characteristic bandwidth can be reported as a 3 dB half-width or a 95% cumulative-energy half-bandwidth; in both cases we observe the collapse BW $\sqrt{p} \approx$ const, consistent with the prediction above.

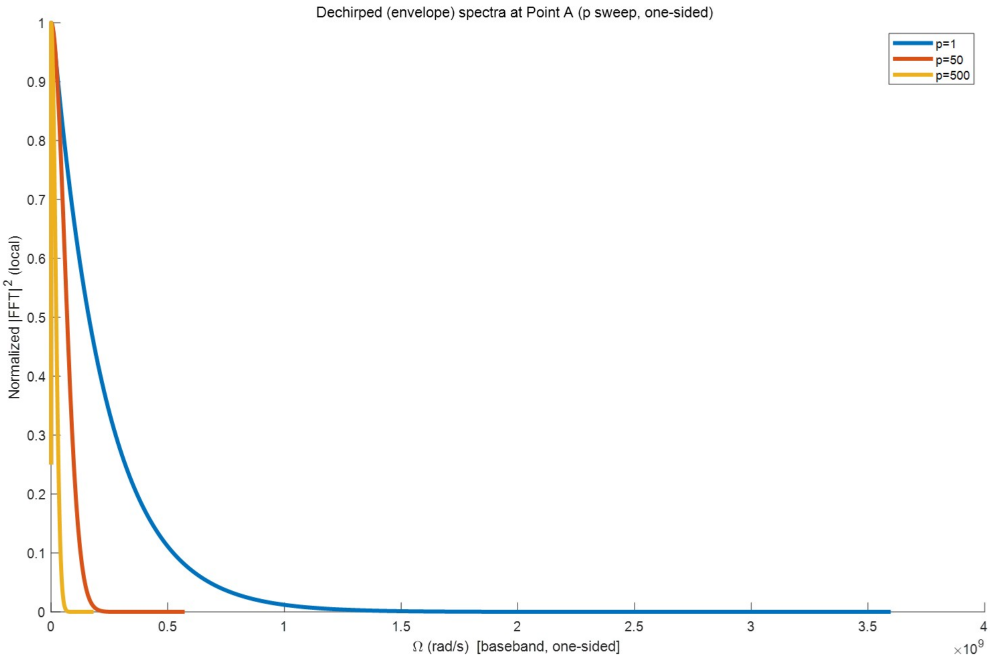

Figure S2 (FD). One-sided, baseband, de-chirped envelope spectra of the azimuthally polarized Flying Donut field at the ring maximum ($z = 0$). Each curve corresponds to a different shape parameter p; the spectra are normalized to unity within the plotted window. As p increases, the spectrum narrows markedly; as p decreases, it broadens.

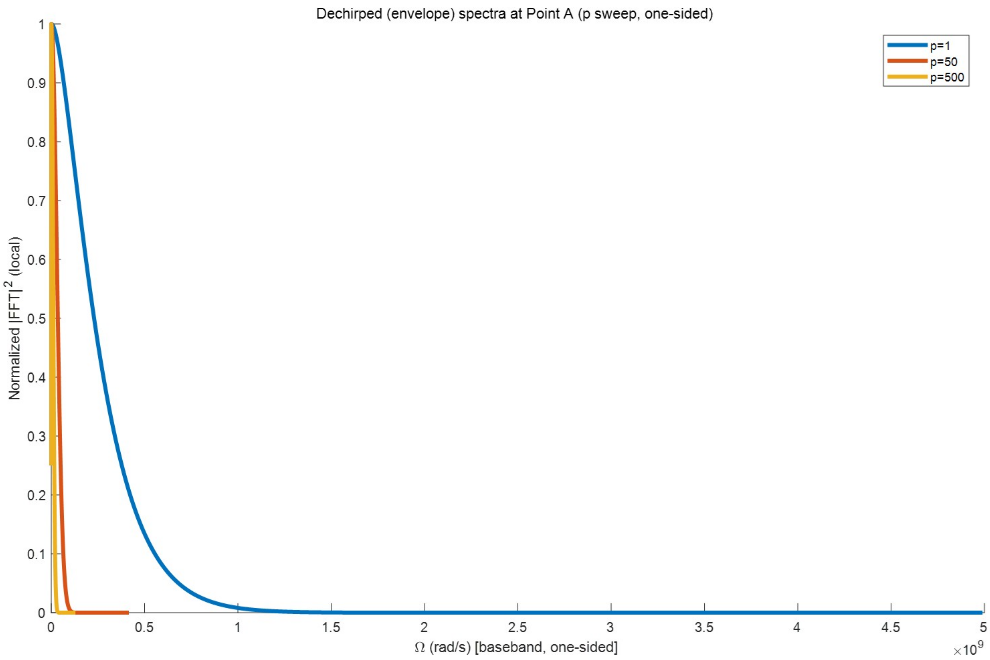

Figure S3 (FP). Same analysis for the Flying Pancake field (evaluated at the transverse position of maximum $|E_\theta|$ at $z = 0$). The same p-trend is observed: larger p produces a narrower envelope spectrum, smaller p a broader one.
